# Gut microbiota and aging: current understanding and future perspectives

**DOI:** 10.1186/s43556-026-00499-0

**Published:** 2026-06-25

**Authors:** Meng Lan, Huiping Ding, Yu Cao, Juxiong Liu, Liqun Tu, Shoupeng Fu, Wenjin Guo

**Affiliations:** 1 State Key Laboratory for Diagnosis and Treatment of Severe Zoonotic Infectious Diseases, Key Laboratory for Zoonosis Research of the Ministry of Education, Institute of Zoonosis, College of Veterinary Medicine, Jilin University, Changchun, 130062 China; 2 Division of Gastroenterology and Hepatology, Department of Medicine, Alway Building, Stanford University, 300 Pasteur Drive, Stanford, CA 94305 USA

**Keywords:** Gut microbiota, Aging, Inflammaging, Gut barrier, Microbiota-based interventions

## Abstract

Aging is a complex biological process characterized by progressive functional decline at molecular, cellular, and systemic levels, accompanied by increased susceptibility to chronic diseases. Accumulating evidence indicates that the gut microbiota plays a critical role in shaping aging trajectories and age-related health outcomes. This review systematically summarizes current research progress on the relationship between gut microbiota and aging. We first describe the characteristic alterations of the gut microbiota during aging, including reduced microbial diversity, shifts in core bacterial taxa, and profound changes in microbial metabolite profiles such as short-chain fatty acids, bile acid derivatives, and tryptophan metabolites. We then discuss the mechanistic links between gut microbiota dysbiosis and age-related functional decline, focusing on immunosenescence and inflammaging, gut barrier dysfunction, metabolic disorders and oxidative stress, as well as endocrine and neuroendocrine regulation through gut–organ axes. In addition, major internal and external factors influencing gut microbiota composition in the elderly, including diet, medication use, lifestyle, host immunity, and living environment, are reviewed. Finally, we summarize current and emerging gut microbiota–targeted anti-aging intervention strategies, such as dietary modulation, probiotics, prebiotics, postbiotics, fecal microbiota transplantation, and natural product–based approaches, and discuss future research directions and clinical translation challenges. Overall, this review highlights the gut microbiota as a key modifiable factor in aging biology and underscores its potential as a promising target for promoting healthy aging.

## Introduction

Aging is the progressive decline of the body's molecules, cells, and functions over time, eventually leading to dysfunction and death [1, 2]. At the molecular level, telomere shortening is considered an important marker of aging. When telomeres become extremely short or damaged, a DNA damage response is activated, leading to telomere dysfunction and triggering cellular senescence or death [3]. At the cellular level, senescent cells exhibit reduced proliferative capacity and cell cycle arrest, accompanied by the production of senescence-associated secretory phenotype (SASP) [3]. Senescent cells release large amounts of pro-inflammatory cytokines and other SASP factors, creating a chronic inflammatory tissue microenvironment that accelerates the aging process [4]. At the functional level, mitochondrial dysfunction is also a common characteristic of aging: accumulation of mitochondrial DNA damage, decreased efficiency of the electron transport chain, and excessive production of reactive oxygen species (ROS) further exacerbate damage to cells and tissues [5]. An emerging factor closely related to the above aging markers is the gut microbiota [6]. The gut microbiota is a complex microbial ecosystem in the human body, mainly composed of bacteria, archaea, fungi, and viruses, with bacteria predominating. Under healthy conditions, the gut microbiota has a mutualistic relationship with the host. It helps break down dietary fiber, synthesize essential vitamins, and produce metabolic products like short-chain fatty acids, providing nutrition and energy to the host [7, 8]. In addition, the gut microbiota participates in the development and functional regulation of the immune system, influencing the host's immune responses and susceptibility to diseases [9]. They also affect the occurrence and progression of host-related diseases through the gut-brain axis, gut-liver axis, and gut-mammary axis [10].

**Fig. 1:**
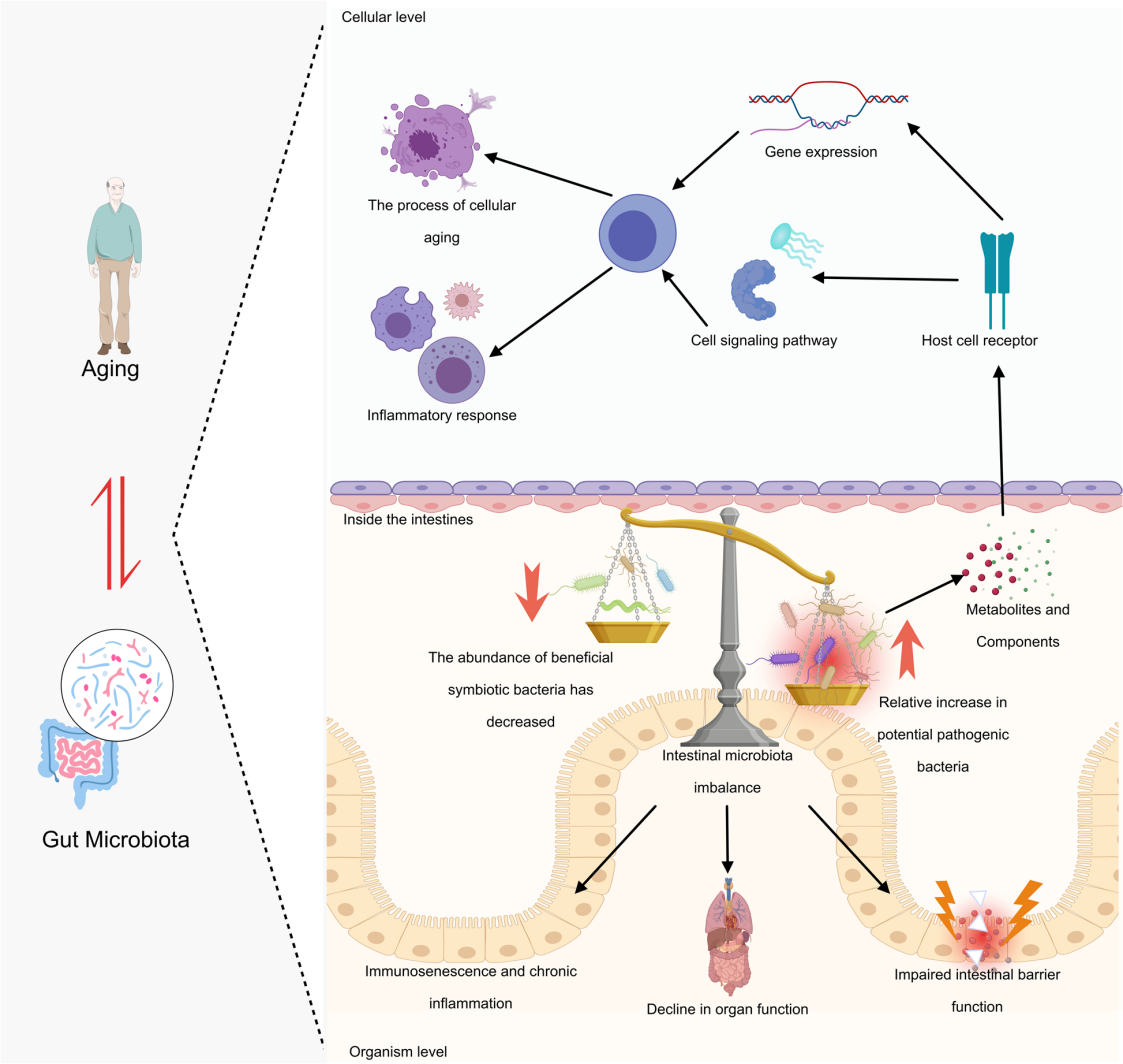
During the aging process, the composition and structure of the gut microbiota change, with an imbalance between beneficial bacteria and potential pathogens, leading to organ dysfunction, immune aging, and inflammation. Moreover, microbial metabolites further influence the host's aging status

A large body of research evidence indicates that the composition and function of the gut microbiota undergo significant changes during aging, and these changes have a bidirectional relationship with aging phenotypes [11, 12].During the aging process, the composition, structure, and function of the gut microbiota change markedly, characterized by a decrease in beneficial symbiotic bacteria and a relative increase in potentially pathogenic bacteria, resulting in an imbalance in the gut microecology [12, 13]. These age-related dysbiosis phenomena have been recognized as an important component of aging biology—in the latest summary of the twelve hallmarks of aging, both chronic inflammation and gut microbiota imbalance are included and closely related [12–15]. Gut microbiota dysbiosis is closely associated with multiple organ functional decline, immunosenescence, increased chronic inflammation levels, and weakened gut barrier function in the elderly, all of which are "signs of aging" [16, 17]. On the other hand, the gut microbiota communicates with host cells through its metabolites and components [18, 19]. For example, microbiota-derived short-chain fatty acids (SCFAs), bile acid derivatives, and tryptophan metabolites like indoles can bind to host cell receptors, modulating host cell signaling pathways and gene expression, thereby affecting inflammatory responses, metabolic states, and even cellular aging processes [19, 20]. In other words, changes in the gut microbiota may both accelerate aging progression and represent promising new intervention targets to delay aging (Fig. 1).

Given the close relationship between the gut microbiota and aging, a deep understanding of the interaction mechanisms between the two is of great significance for revealing the nature of aging and seeking anti-aging intervention strategies [21]. This review aims to systematically summarize the current research progress on the relationship between gut microbiota and aging, including characteristic changes in gut microbiota during aging, how dysbiosis affects body aging through various mechanisms, and possible intervention and treatment strategies.

## Characteristic changes of gut microbiota during the aging process

With aging, the human gut microbiota undergoes characteristic changes in diversity, core microbiota composition, and metabolic functions [6, 22, 23]. This section will overview the main changes in the gut microbiota of elderly individuals.

### Changes in α diversity of gut microbiota in the elderly and its association with frailty phenotype

**Fig. 2:**
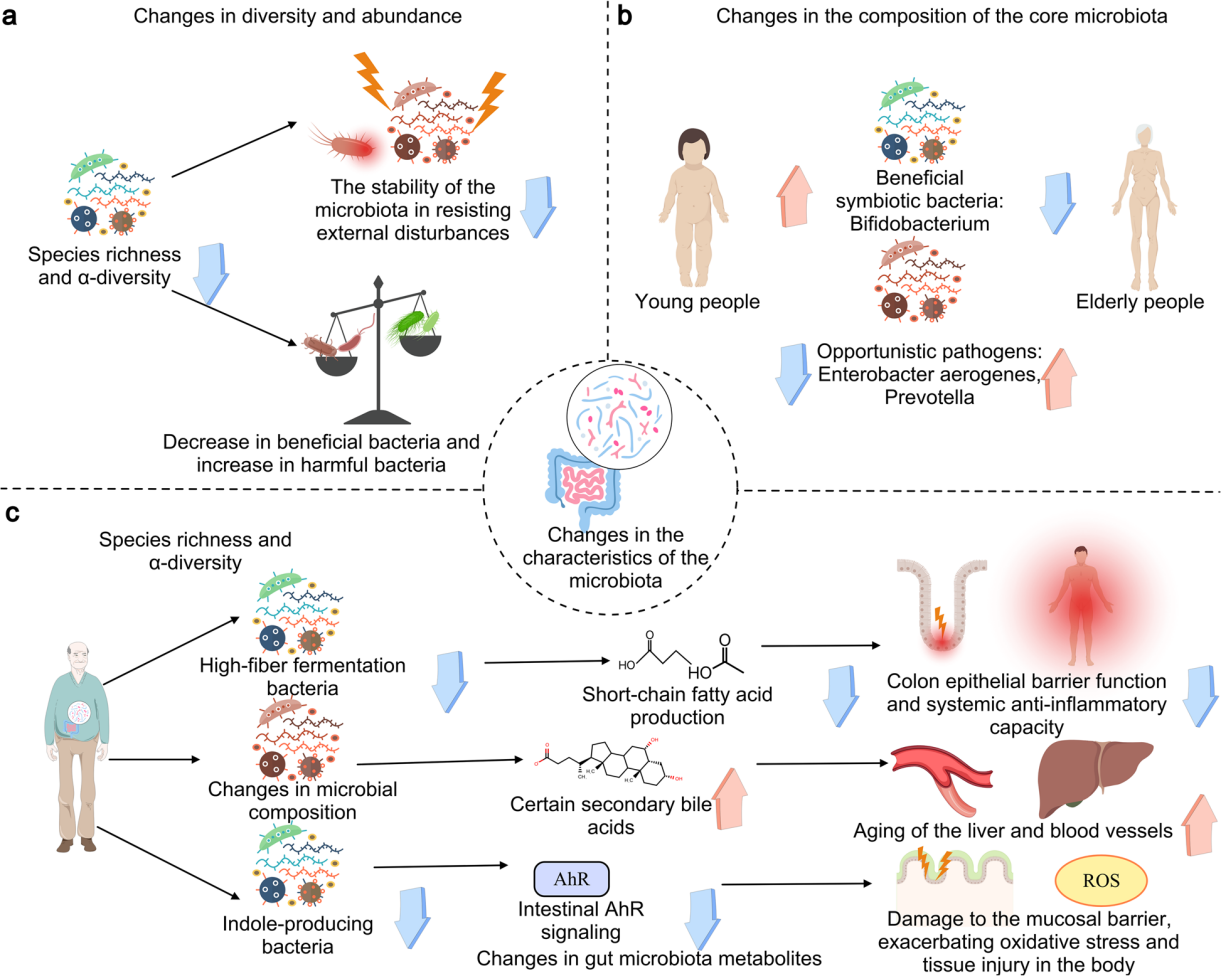
(**a**) The richness and diversity of the gut microbiota in elderly people change. (**b**) The composition of the core gut microbiota changes during aging, with alterations in the abundance of beneficial symbiotic bacteria and potential pathogenic bacteria. (**c**) The metabolites produced by the gut microbiota in the aging state change, thereby affecting gut function. ROS: reactive oxygen species; AhR: Aryl hydrocarbon receptor; NF-κb: nuclear factor kappa-B

The diversity of the gut microbiota is often regarded as an important indicator of gut health. Numerous studies have shown that, compared to young people, the species richness and alpha diversity of gut microbiota in elderly people are generally reduced. For example, a study on adults of different ages found that the microbial diversity in the healthy elderly group was significantly lower than that in the middle-aged and young groups [24]. A decrease in gut ecological diversity implies poorer stability of the microbial community in resisting external disturbances and is often accompanied by an imbalance characterized by a reduction in beneficial bacteria and an increase in harmful bacteria [25]. However, there is some controversy regarding changes in microbial diversity in the elderly. Some studies have observed that in very old healthy individuals, gut microbiota diversity does not significantly decrease; individual reports have even found that certain centenarians have higher gut microbiota diversity than middle-aged people [26, 27]. Some scholars speculate that this may be due to weakened immune control over the microbiota by some high-aged hosts, allowing more bacterial species to coexist. Nevertheless, overall, "microbiome aging" typically manifests as a decline in diversity and is considered to be associated with frailty and weakening phenotypes [11]. For instance, in frail elderly individuals, gut microbiota diversity is significantly reduced, and their microbial community composition tends to shift toward a disadvantageous state, which may further impair health [28] (Fig. 2a).

### Changes in the composition of core microbial communities

In addition to overall diversity, aging is also accompanied by changes in the relative proportions of the core gut microbiota. At the phylum level, studies have found a reorganization of the dominant bacterial populations in the guts of elderly people. For example, the proportion of Bacteroidetes often increases in the elderly group, while the ratio of Firmicutes relatively decreases [6, 29]. Meanwhile, some Gram-negative Proteobacteria are more enriched in the guts of older adults [30, 31]. Proteobacteria include endotoxin-producing bacteria such as Escherichia coli, and their increase is often regarded as a marker of dysbiosis. This pattern of "reduced beneficial bacteria and increased potentially harmful bacteria" makes the gut microbiota of elderly people more prone to pro-inflammatory and pathogenic tendencies [14, 24]. For instance, beneficial symbiotic bifidobacteria are usually abundant in the intestines of infants and adults, but their abundance significantly decreases in the elderly, especially those over 80 years old [11]. The reduction of bifidobacteria may weaken the gut’s fermentation metabolism and epithelial protective functions. Conditioned pathogenic bacteria such as Enterobacter aerogenes and Prevotella increase in proportion in the aged gut, producing endotoxins and harmful metabolites that potentially threaten host health [14, 31]. It should be noted that some studies focus on the microbiota characteristics of long-lived populations. The gut microbiota of centenarians appears to have features different from those of general elderly individuals [32]. Research has reported higher abundances of mucin-degrading bacteria like Akkermansia and certain butyrate-producing Clostridiales, as well as traditionally beneficial bifidobacteria, in the feces of long-lived elders [19, 26, 32]. These bacteria might contribute to the host’s healthy longevity by strengthening the gut mucosal barrier and suppressing gut pathogens [32]. Therefore, the alteration of the core gut microbiota composition is one of the important hallmarks of aging, and different microbiota composition patterns may correspond to different qualities of aging.

It is noteworthy that gut microbiota dysbiosis has been recognized as a key feature and contributing factor of organismal aging. Dysbiosis specifically includes phenomena such as reduced diversity, decreased symbiotic bacteria, and overgrowth of opportunistic pathogens mentioned above. This imbalanced state triggers a series of negative chain reactions, such as an increase in harmful microorganisms and their metabolites, and disruption of microecological competition, which lays the groundwork for subsequent inflammation and metabolic disorders. In summary, changes in microbiota composition associated with aging provide clues to understanding why the elderly are more susceptible to diseases; the loss of probiotics and the proliferation of pathogenic bacteria may be among the causes of multi-system functional decline in the body (Fig. 2b).

### Gut microbiota SCFA decrease and changes in bile acid and tryptophan metabolic pathways

The gut microbiota influences host physiology by producing various metabolites. Among them, short-chain fatty acids (SCFAs), bile acid derivatives, and tryptophan metabolites play important roles in maintaining host metabolic and immune homeostasis [19, 20, 33]. Age-related changes in the microbiota are accompanied by broad alterations in the profile of these key metabolites [20, 34].

Firstly, the production of short-chain fatty acids (SCFAs) tends to decrease in the intestines of elderly people [19]. SCFAs are mainly produced by gut symbiotic bacteria fermenting dietary fiber, including acetate, propionate, and butyrate [35, 36]. Butyrate is the primary energy source for colonic epithelial cells and exerts anti-inflammatory and epithelial-supporting functions. In older adults, reductions in major SCFAs and in butyrate-associated fermentative capacity have been reported, together with age-related loss of beneficial butyrate-producing taxa [19, 34]. The decrease in butyrate-producing bacteria is believed to weaken colon epithelial barrier function and systemic anti-inflammatory capacity [34, 37]. SCFA deficiency may therefore contribute to more pronounced chronic low-grade inflammation in the elderly body. In summary, with aging, the gut microecology’s ability to provide beneficial metabolites declines, leading to a relative deficiency in energy-supporting and immune-regulating signals needed by host cells [34].

Secondly, the metabolism of bile acids by gut bacteria also changes with age [34, 38]. Primary bile acids entering the intestine are further converted by the gut microbiota into secondary bile acids, and aging-associated dysbiosis can disturb this conversion pattern [34, 39]. In aging mice, gut microbiota remodeling can partly reverse dysregulated systemic bile acid homeostasis, indicating a direct link between age-related microbiota shifts and bile acid perturbation [34]. Excess bile acids, including secondary species such as deoxycholic acid (DCA) and lithocholic acid (LCA), can disturb epithelial barrier-related signaling and promote inflammatory injury in experimental systems [19, 34]. Moreover, enhanced gut bile acid absorption and altered bile acid profiles have been linked to neuroinflammation and cognitive decline in aging mice and in elderly-related cognitive impairment, while bile acid sequestration alleviates these phenotypes [38]. Mechanistically, bile acid receptor pathways, including FXR/TGR5 signaling, provide a plausible route through which microbiota-dependent bile acid dysregulation can reshape host metabolic and inflammatory responses [34, 40–42].

In addition, tryptophan metabolites and other microbial metabolites are also worth attention in aging [20, 33]. The gut microbiota can metabolize tryptophan into indole and its derivatives, which act on host pathways and participate in regulating gut immunity and epithelial homeostasis [33, 43, 44]. In vivo and disease-model studies further demonstrate that microbiota-derived tryptophan metabolites can restrain inflammatory responses via host receptor pathways such as AhR [45, 46]. Conversely, metabolomic analyses in aging mice show that multiple circulating metabolites change with age, including marked alterations in tryptophan-related microbial co-metabolites, consistent with broad remodeling of microbe-host co-metabolism in older organisms [20]. In summary, gut microbiota functions become disordered with aging, and the profile of metabolic products changes—beneficial metabolites decline while maladaptive metabolic patterns increase—acting both as a marker of microbial aging and a potential mediator that may trigger host aging [20].

It is worth mentioning that changes in gut microbial metabolites are not limited to local effects within the gut but can influence multiple distant organs through systemic circulation [20]. For example, aging-associated defects in microbiota/butyrate signaling have been linked to gut inflammation, barrier dysfunction, and impaired brain function [34]. Similarly, plasma levels of trimethylamine N-oxide (TMAO), a metabolite produced by gut microbiota from dietary choline, tend to be elevated with aging, and high TMAO has been shown to be associated with endothelial dysfunction, vascular oxidative stress, and cardiovascular aging risk [45]. Therefore, understanding the alterations in aging-associated microbial metabolites is crucial for elucidating how gut microbiota systemically affect aging [20] (Fig. 2c).

In summary, the gut microbiota undergoes a series of characteristic changes during the aging process, including a decline in microbial diversity, an imbalance in the core microbiota structure, and alterations in the metabolite profile. These changes are both concomitant phenomena of aging and potential factors that promote aging. In the next section, we will discuss how dysbiosis of the gut microbiota interacts with multiple body systems through various mechanisms, triggering or accelerating age-related functional decline.

## Mechanisms linking gut microbiota dysbiosis with age-related functional decline

### Immunosenescence and inflammatory aging

**Fig. 3:**
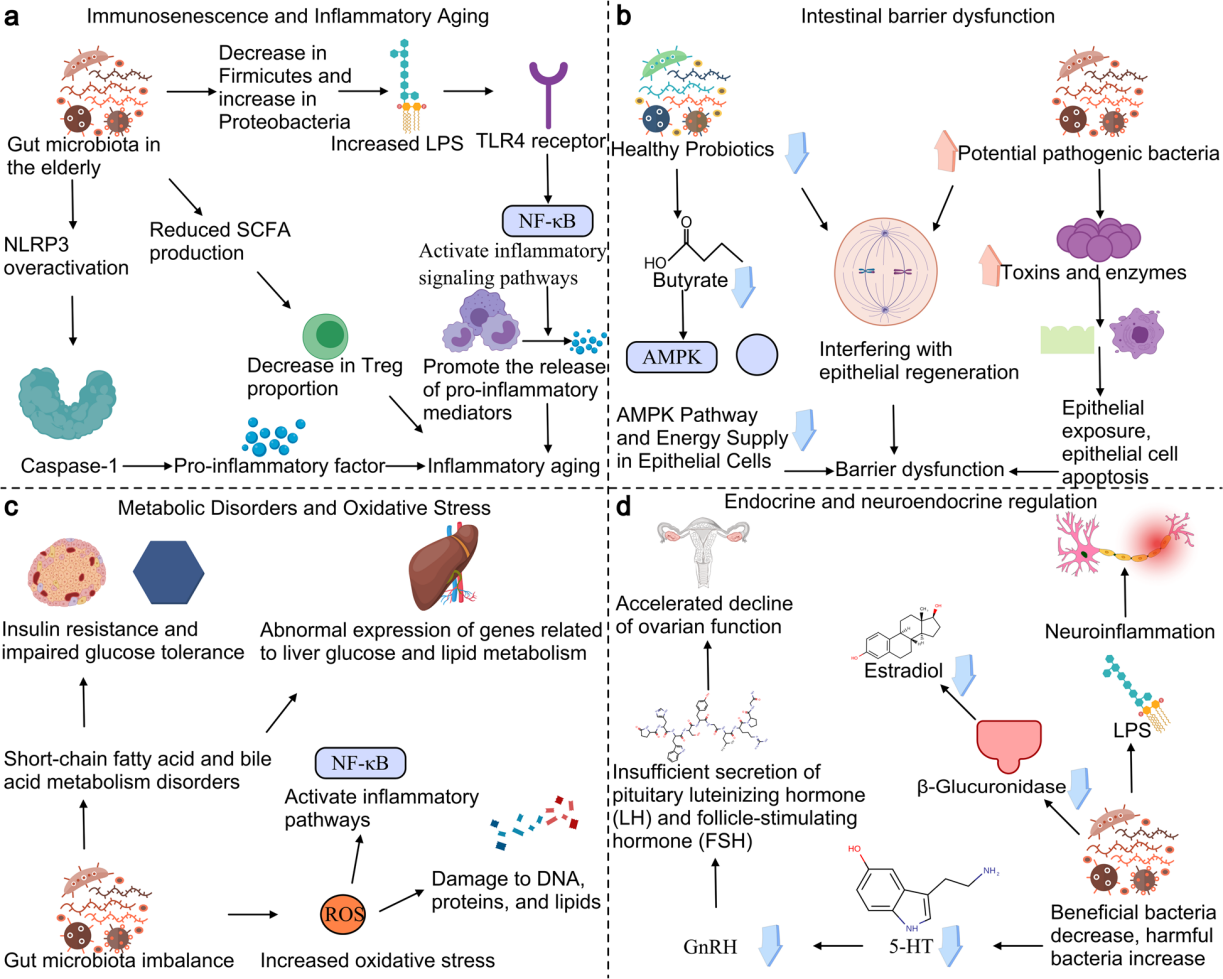
(**a**) The mechanism by which gut microbiota imbalance leads to immune aging and inflammatory aging. (**b**) The mechanism by which changes in gut microbiota composition cause gut barrier dysfunction. (**c**) The mechanism by which alterations in gut microbiota lead to metabolic disorder and oxidative stress. (**d**) The mechanism of gut microbiota imbalance at the endocrine and neuroendocrine levels

Immunosenescence refers to the progressive decline in immune system function with increasing age, including changes in both innate and adaptive immunity [34, 47, 48]. Elderly individuals often exhibit changes in the number and function of immune cells, such as reduced T cell diversity, diminished antibody production by B cells, and dysfunction of macrophages and neutrophils [49–51]. At the same time, older adults show elevated systemic chronic low-grade inflammation, known as "inflammaging" [52]. Inflammaging is characterized by increased baseline levels of pro-inflammatory cytokines in the blood, such as IL-6, TNF-α, and CRP [53, 54]. It is interrelated with immunosenescence and together forms an important underlying factor for the high incidence of diseases in the elderly (Fig. 3a).

Recent studies have revealed that dysbiosis of the gut microbiota is a key driver of immunosenescence and inflammatory aging [55]. Firstly, changes in the microbiota can lead to increased endotoxin load. Aging is accompanied by a decrease in Firmicutes and an increase in Proteobacteria, the latter possessing large amounts of lipopolysaccharides (LPS) on their surfaces. When gut barrier function is weakened, LPS more easily passes through the gut wall into the bloodstream. Elevated levels of LPS, along with LPS-binding protein (LBP) and its receptor, are often detected in the serum and tissues of elderly individuals, all of which are markers of gut-derived endotoxin translocation. As a potent immune adjuvant, LPS can activate inflammatory signaling pathways such as NF-κB through the TLR4 receptor, stimulating macrophages and monocytes to release pro-inflammatory mediators like tumor necrosis factor-alpha (TNF-α) and interleukin-6 (IL-6), thereby inducing systemic chronic inflammation [22]. Therefore, endotoxemia caused by gut microbiota dysbiosis is considered an important cause of inflammatory aging in the elderly.

Secondly, changes in microbial metabolites also affect the balance of immune cells [56–58]. For example, short-chain fatty acids (SCFAs) are key mediators connecting the microbiota with immunity. SCFAs such as butyrate and propionate can act on G protein-coupled receptors on the surface of immune cells or enter the nucleus to inhibit histone deacetylases, exhibiting overall anti-inflammatory and immunoregulatory effects [59, 60]. Studies have shown that SCFAs promote the polarization of macrophages toward the anti-inflammatory M2 phenotype and inhibit the activation of pro-inflammatory M1 macrophages; meanwhile, butyrate can induce dendritic cells to produce inhibitory molecules and promote the differentiation of peripheral regulatory T cells (Tregs) [8]. Treg cells are important for maintaining immune homeostasis and can secrete anti-inflammatory factors such as IL-10 and TGF-β. In elderly individuals, due to a reduced capacity of the microbiota to produce SCFAs, the proportion of Tregs often decreases, leading to a loss of control over inflammation, causing Th17 imbalance and exacerbating tissue-damaging inflammation. Moreover, microbial metabolites can also influence myeloid cell senescence [61].

Furthermore, dysbiosis triggers chronic activation of innate immune sensors. Overactivation of innate immune sensing complexes such as the NOD-like receptor protein 3 (NLRP3) inflammasome is commonly detected in aging tissues. NLRP3 is an intracellular pattern recognition receptor that can be activated by pathogen-associated molecular patterns or damage-associated molecular patterns. Activated NLRP3 inflammasomes promote Caspase-1 cleavage to mature pro-inflammatory factors like IL-1β and IL-18, and induce pyroptosis, an inflammatory cell death, in macrophages and other cells. Due to declined gut barrier function in the elderly, LPS, peptidoglycan, and other molecules from the gut continuously stimulate NLRP3 inflammasomes in liver Kupffer cells and cardiac macrophages [62]. For example, in an aging-related atrial fibrillation model, gut dysbiosis was found to activate the NLRP3 inflammasome in atrial tissue through LPS and metabolic imbalance, promoting atrial structural remodeling and arrhythmia. Similarly, persistent NLRP3 activation and IL-1β release have been observed in non-alcoholic steatohepatitis and aging arteriosclerosis, leading to inflammatory damage in hepatocytes and vascular endothelium This “silent chronic inflammation,” mediated by innate immune sensors, is involved in various aging pathological processes [13].

Finally, it is necessary to mention that the aging changes in immune cell composition and function are partly driven by the microbiota [63]. For example, in the elderly, thymic atrophy and reduced T cell neogenesis occur, but effector T cells remain in a state of chronic activation under continuous stimulation by gut microbiota antigens; this phenomenon is called "bystander antigen-driven." Chronic antigen stimulation can cause T cell exhaustion and abnormal differentiation, thereby weakening immune surveillance against new infections and tumors. Additionally, the decline in B cell function and the increase in low-affinity antibodies in the elderly are also related to continuous antigen stimulation at mucosal sites such as the gut. Thus, gut microbiota dysbiosis exacerbates immune aging through multiple mechanisms, both by "adding fuel to the fire" to increase inflammatory burden and by "overdrawing life" to cause immune cell overwork and exhaustion. Immunosenescence and inflammaging further lead to chronic tissue damage and reduced regenerative capacity, which are considered common backgrounds for various aging-related diseases.

In summary, gut microbiota dysbiosis is an important driving factor of immunosenescence and inflammaging. Inhibiting chronic inflammation driven by microbiota is considered one of the potential strategies to delay aging and prevent chronic diseases in the elderly.

### Gut barrier dysfunction

The gut barrier, composed of epithelial cells, mucus layer, and junctional complexes, is the first line of defense against the invasion of harmful substances and maintaining internal homeostasis [64]. Under normal conditions, the tight junctions between gut epithelial cells are tightly sealed, preventing gut contents from easily passing through the epithelium into systemic circulation [65–67]. However, the aging process causes progressive weakening of the structure and function of the gut barrier, a phenomenon metaphorically called "leaky gut" [68]. This manifests as a reduction in the number of goblet cells in the gut mucosa, decreased and diluted mucus secretion leading to thinning of the mucus barrier [69, 70];slowed epithelial cell renewal, increased permeability, and decreased expression of tight junction proteins [71]; and weakened immune defense functions of the gut lamina propria. These changes increase the permeability of the gut wall, allowing bacteria and their products to enter the body through "gut mucosal translocation," triggering chronic inflammation and organ damage (Fig. 3b). [72, 73]

Changes in the gut microbiota play a key role in gut barrier dysfunction. Firstly, a reduction in symbiotic bacteria deprives the barrier of nutrients and stimulatory sources [72]. Healthy symbiotic bacteria such as Bifidobacteria and butyrate-producing bacteria generate butyrate through fermentation, which can directly provide energy to gut epithelial cells and promote mucus secretion and tight junction protein expression in the gut epithelium [72, 74]. At the same time, normal symbiotic bacteria can interact with epithelial and immune cells, inducing the secretion of protective antibodies such as IgA, thereby enhancing barrier function [75]. However, in the elderly, butyrate production decreases, epithelial cell energy supply is insufficient, and the lack of butyrate activation of the AMPK pathway leads to impaired assembly of tight junction proteins, making the barrier more "loose" [72, 76]. Studies in mice have confirmed that a reduction of butyrate-producing bacteria in the intestines of aged mice leads to decreased signaling of free fatty acid receptors FFAR2/3, reduced mucus production by goblet cells, ultimately resulting in increased mucosal permeability and elevated inflammation; supplementation with butyrate can partially reverse these changes and restore gut barrier function. This indicates that the deficiency of beneficial gut metabolites is one of the important causes of barrier dysfunction [74].

Secondly, the increase of potential pathogenic bacteria can damage the barrier structure [65]. Certain Gram-negative bacteria enriched in the microbiota of the elderly can produce toxins and enzymes that directly destroy the mucosal barrier [77]. For example, Escherichia coli and Shigella can secrete proteases that break down the mucus layer, exposing the epithelium [78, 79]; Clostridium perfringens and others can produce toxins that induce epithelial cell apoptosis [80]. In addition, the excessive colonization of pathogenic bacteria competes with epithelial cells for nutrients and triggers the release of inflammatory mediators by epithelial cells, causing chronic latent inflammation in the intestines, which further weakens barrier integrity [81]. Common conditional pathogenic bacteria in the elderly gut have been found to be positively correlated with gut permeability and systemic inflammation markers. Therefore, the expansion of harmful bacteria caused by dysbiosis attacks the barrier through multiple pathways, leaving it riddled with defects.

Furthermore, the negative impact of the aging microbiota on the gut barrier is also reflected in the disruption of epithelial regeneration [82]. When young, gut epithelial cells are renewed every 3–5 days, with new cells provided by the proliferation and differentiation of basal stem cells. In elderly individuals, the function of gut epithelial stem cells declines, and regenerative capacity weakens [83]. Recent studies indicate that certain metabolites, such as lithocholic acid, increase in the gut microbiota of aged mice and inhibit epithelial stem cell proliferation [84], while the reduction of beneficial bacteria leads to decreased regenerative signals like epidermal growth factor [74, 75, 78]. Conversely, transplanting the microbiota from young mice into aged mice can enhance stem cell activity in the gut crypts of old mice, promoting epithelial regeneration and thereby strengthening gut wall integrity. This suggests that the composition of the gut microbiota is crucial for maintaining epithelial renewal in the elderly [68].

Due to gut barrier dysfunction, gut-derived infections and inflammation are more likely to occur in the elderly. For example, older adults are more prone to bacteremia caused by bacterial translocation and gut-derived infectious diseases; even in the absence of infection, barrier breaches may allow continuous entry of microbial products, triggering systemic immune activation and chronic inflammation. Notably, studies have found that elevated blood levels of LPS correlate with the severity of frailty syndrome in the elderly. Endotoxins such as LPS not only induce inflammation but also directly act on Toll-like receptors on the surface of distal organ cells, causing insulin resistance and functional decline of tissues. For instance, LPS-induced disruption of insulin signaling may be one reason for the high prevalence of type 2 diabetes in older adults; LPS activation of brain microglia may exacerbate neuroinflammation and cognitive decline.

In summary, gut microbiota imbalance weakens gut barrier function, causing "leaky gut" and systemic chronic endotoxin exposure, thereby promoting multi-system aging. Maintaining and repairing the gut barrier in the elderly is considered one of the important ways to improve their health. The role of the gut barrier in aging also suggests that the gut could be viewed as a focal organ for anti-aging interventions, and "treating the gut" might be able to "nurture the body."

### Microbiota metabolite-mediated metabolic disorders and oxidative stress

The aging process is accompanied by a decline in systemic metabolic regulation capacity and an increase in oxidative stress levels. Increasing evidence suggests that gut microbiota dysbiosis can accelerate aging-related metabolic disorders by affecting host metabolic pathways and redox balance [85] (Fig. 3c).

Firstly, changes in the gut microbiota can lead to imbalances in host energy metabolism [86–88]. In young and healthy conditions, the gut microbiome participates in various metabolic processes of the host, including carbohydrates, fats, and amino acids, providing additional energy and regulating nutrient utilization efficiency [89]. As age increases, alterations in the composition and function of the microbiota disrupt these processes [90, 91]. For example, the aforementioned reduction in SCFAs not only affects the local gut environment but also impacts systemic energy metabolism and appetite regulation [92]. Butyrate and propionate can reach the liver and muscles through the bloodstream, activating the AMPK pathway to promote fatty acid oxidation and improve insulin sensitivity [37, 93]. However, in elderly people, the reduced capacity of the microbiota to produce SCFAs leads to insufficient AMPK activation [94]; combined with the presence of chronic inflammation, this often results in insulin resistance and impaired glucose tolerance [93]. Additionally, changes in bacterial species involved in bile acid metabolism within the gut microbiota of elderly individuals cause disturbances in the bile acid pool composition, subsequently affecting cholesterol and lipid metabolism in the liver and throughout the body [95]. Some studies have found that aged mice with dysbiotic microbiota show decreased plasma primary bile acids and accumulation of secondary bile acids, which interferes with receptor signals such as FXR and TGR5, leading to abnormal expression of hepatic genes related to glucose and lipid metabolism [34, 95]. These metabolic disorders align with the susceptibility of elderly individuals to metabolic diseases like fatty liver and atherosclerosis [96]. Furthermore, the gut microbiota participates in methylamine metabolism through enzyme production; certain gut bacteria metabolize dietary choline and carnitine into trimethylamine (TMA), which is oxidized in the liver to trimethylamine TMAO [45, 97]. Research indicates that TMAO levels in humans and mice increase with age, with elderly individuals showing significantly higher circulating TMAO than the young, correlating with elevated blood pressure and atherosclerosis markers [96]. TMAO promotes macrophage accumulation and foam cell formation in arterial walls, accelerating vascular aging [98]. In animal experiments, aged rats treated long-term with high TMAO exhibited vascular endothelial dysfunction, reduced elasticity, and other aging phenotypes [99, 100]. Moreover, elevated TMAO has been linked to cognitive decline, possibly by exacerbating neuroinflammation and oxidative stress [101]. This evidence suggests that metabolites produced by dysbiotic microbiota, such as TMAO, are important inducers of aging-related metabolic abnormalities [102, 103].

Secondly, gut microbiota imbalance significantly affects the host's redox balance, leading to enhanced oxidative stress [104]. Oxidative stress refers to a state in which the production of reactive oxygen species and free radicals exceeds the body's antioxidant capacity, causing damage to biomolecules. In aging tissues, ROS levels often increase, and dysregulated microbiota may be an important driver of ROS overproduction [105]. On one hand, microbiota imbalance sustains chronic inflammation and immune activation, thereby amplifying ROS generation and promoting oxidative injury beyond the gut [104]. On the other hand, fecal microbiota transplantation studies have directly shown that aged donor microbiota can transmit oxidative-stress phenotypes to young recipients, increasing oxidative stress and contributing to functional decline in recipient tissues, including the brain [104, 105]. Additionally, certain microbiota-associated substances can also induce ROS production. For example, the endotoxin lipopolysaccharide (LPS) not only triggers inflammation but also directly impairs mitochondrial function and enhances ROS generation in endothelial and cardiomyocyte cells [106, 107]. Moreover, a recent aging study identified the advanced glycation end product Nε-carboxy methyl lysine (CML) as a microbiota-dependent metabolite that accumulates in the aging context and induces ROS burst, mitochondrial dysfunction, and ATP depletion in microglia [107]. Research has also shown that aged gut microbiota-related oxidative stress is accompanied by disruption of antioxidant signaling involving the OLA1-Nrf2 axis, which may increase host vulnerability to oxidative damage. Excessive ROS damages DNA, proteins, and lipids, which is a key mechanism driving cellular senescence and death [59]. At the same time, ROS can act as signaling molecules to activate inflammatory pathways, further exacerbating the vicious cycle of inflammation and oxidative stress [106]. In the cardiovascular system, this may manifest as arrhythmia susceptibility and other forms of oxidative-stress-related cardiac dysfunction [107]; in the nervous system, it may contribute to synaptic dysfunction, cognitive decline, and greater susceptibility to neurodegenerative change [59].

Lastly, it is worth mentioning that the gut microbiota may also influence host metabolism and oxidative stress through epigenetic regulation and cellular signaling. For example, the microbial metabolite butyrate is a histone deacetylase (HDAC) inhibitor that can reshape host gene-expression programs [59]. In addition, microbiota-derived metabolites can modulate signaling networks closely related to aging and stress responses, including mTOR-, AMPK-, and NF-κB-related pathways, thereby regulating cellular metabolism, inflammation, and survival programs at a deeper level [59]. For instance, experiments have found that administering certain gut microbiota metabolites to worm models can extend their healthy lifespan by activating mitochondrial function and antioxidant stress pathways. These epigenetic and signaling effects suggest that the gut microbiota may have long-term impacts on the metabolic and antioxidant gene networks at the host cellular level, which warrants further investigation.

Overall, dysbiosis of the gut microbiota induces host metabolic disorders and oxidative stress through multiple mechanisms, including imbalance in nutrient supply and metabolic signaling, as well as direct damage caused by inflammation and ROS. These factors collectively drive the aging process of the organism.

### Endocrine and neuroendocrine regulation

The influence of the gut microbiota is not limited to the immune and metabolic fields; it also affects the aging of multiple host organ systems through endocrine and neuroendocrine pathways. Here, we focus on the role of the gut microbiota in regulating gonadal axis hormones and the gut-brain axis, as well as its potential impact on reproductive and neurological aging (Fig. 3d).

Firstly, recent attention has been given to research on how the gut microbiota influences reproductive endocrine function through the gut-brain-gonadal axis. In female aging (including natural menopause and pathological premature ovarian insufficiency, POI), evidence has emerged linking changes in the gut microbiota with alterations in gonadal axis function. Gut microorganisms can produce or regulate various neurotransmitters, one of which is serotonin (5-HT). 5-HT is an important neurotransmitter in the central nervous system and also participates in the regulation of hypothalamic gonadotropin-releasing hormone (GnRH) [91]. Various gut bacteria can synthesize 5-HT or affect 5-HT production by enterochromaffin cells in the gut mucosa; for example, genera such as Streptococcus, Enterococcus, Escherichia, and Bifidobacterium have been confirmed to have the ability to metabolize tryptophan to produce 5-HT. During ovarian aging, studies have observed a relative increase in bacteria with strong 5-HT inactivation or degradation abilities and a decrease in beneficial bacteria like Bifidobacterium. This may lead to a reduction in the total amount of peripheral bioavailable 5-HT. Changes in central 5-HT levels directly affect the firing frequency of GnRH neurons and the pulsatile release of GnRH. Animal experiments have shown that reducing hypothalamic 5-HT signaling results in decreased GnRH synthesis, insufficient secretion of pituitary luteinizing hormone (LH) and follicle-stimulating hormone (FSH), leading to stunted ovarian follicle development, a reduction in corpora lutea, and accelerated decline of ovarian function. In other words, dysregulation of the gut microbiota-5-HT-HPO axis may be one of the contributing factors to premature natural menopause and POI [91, 108]. Studies on patients with idiopathic POI also support this view; their gut microbiota often exhibits dysbiosis characterized by reduced short-chain fatty acid (SCFA)-producing bacteria and increased specific pathogenic bacteria. These patients often accompany very low serum estrogen levels and insufficient GnRH and LH secretion, which are temporally correlated with changes in the microbiota [109]. Although the causal role of microbiota alterations in POI remains undetermined, it is speculated that the lack of beneficial metabolites may induce local ovarian inflammation and metabolic deterioration, increasing follicular apoptosis. We mentioned earlier that butyrate has anti-inflammatory effects; the absence of butyrate may keep the ovarian microenvironment in a chronic inflammatory state, unfavorable for follicle survival and growth. In summary, the gut microbiota impacts the gonadal axis through neurotransmitters and metabolic pathways, providing new insights into understanding female reproductive aging and suggesting that improving gut microbiota might help maintain ovarian function.

Secondly, the gut microbiota can regulate systemic hormone levels, especially the metabolism of sex hormones, referred to as the "gut-estrogen axis" [110]. Estrogens in the human body are metabolized in the liver and excreted as conjugated forms through bile and urine. However, β-glucuronidase produced by the gut microbiota can hydrolyze conjugated estrogens back into free forms, allowing their reabsorption into the bloodstream and thus influencing estrogen levels in the body [108, 111]. This bacterial gene function cluster that regulates estrogen circulation is called the "estrobolome." In young women, a normally functioning estrobolome maintains the dynamic balance of estrogen enterohepatic circulation; whereas in postmenopausal women or some patients with premature ovarian insufficiency (POI), abnormal estrobolome activity may lead to a significant decline in endogenous estrogen levels [109]. For example, reports have noted that β-glucuronidase activity in the feces of POI patients is significantly reduced, suggesting that estrogens that should be reabsorbed are instead directly excreted from the intestine, further reducing systemic E2 levels. Declining estrogen levels lead to a series of aging-related changes, such as osteoporosis, accelerated skin aging, and increased cardiovascular risk. Therefore, the gut microbiota plays a certain role in female systemic aging by affecting estrogen metabolism. Conversely, exogenous estrogen therapy also impacts the microbiota; after HRT use in POI patients, gut microbiota diversity and the abundance of specific bacteria partially recover. This highlights the bidirectional interaction between the microbiota and estrogen. For males, the reciprocal influence between gut microbiota and androgens has also begun to attract attention, though the mechanisms remain unclear.

Third, the gut-brain axis is an important pathway through which the microbiota affects the aging of the nervous system [112–114]. The gut microbiota can communicate bidirectionally with the brain via the vagus nerve, immune pathways, and metabolic routes [112, 115, 116]. As age increases, this axial communication may become abnormal [117]. For example, chronic low-grade inflammation commonly accompanying the elderly can cross the blood–brain barrier and bring inflammatory mediators into the central nervous system, creating a micro-inflammatory environment in brain tissue [118]. Gut bacterial leakage allowing bacterial metabolites such as LPS to enter circulation has also been found to be related to the occurrence of neurodegenerative diseases like Alzheimer's disease [119, 120]. One study transplanted the gut microbiota of aged mice into young germ-free mice, and the recipient mice not only developed gut inflammation but also induced neuroinflammation and decreased neuronal plasticity in the hippocampus, exhibiting memory decline and anxiety-like behavior [72]. This suggests that certain products contained in the aged microbiota may penetrate the blood–brain barrier and damage the brain [113]. Correspondingly, transplantation of young mice microbiota into aged mice showed some cognitive improvement, supporting the idea that the microbiota has a plastic influence on central aging [121]. Additionally, the previously mentioned 5-HT also mediates part of the gut-brain axis function—the gut microbiota-regulated 5-HT not only affects hypothalamic gonadotropin-releasing hormone secretion but also acts on the brain-gut neural circuit, impacting mood and cognition [122, 123]. In summary, the gut microbiota participates in the aging process of the nervous system through multiple neuroendocrine pathways.

Overall, gut microbiota imbalance is closely linked to aging at both the endocrine and neuroendocrine levels. It may accelerate reproductive system aging by affecting gonadal axis hormone levels, as well as alter central nervous system status through the gut-brain axis. Future in-depth research into these mechanisms will help us develop microbiota regulation strategies targeting aging of the gonadal and nervous systems, such as using probiotics to improve mood and cognition, and delaying menopause-related aging by modulating estrogen groups. In the following section on intervention strategies, we will also discuss some preliminary attempts.

### Multidimensional mechanistic crosstalk and cascading amplification mediated by the gut microbiota

Rather than regarding immunosenescence, gut barrier dysfunction, metabolic disturbance, and gut-brain/neuroendocrine alterations as isolated mechanisms of aging, they should be understood as interconnected and progressively amplifying processes within a continuous network driven by gut microbiota dysbiosis [13, 124]. The gut microbiota is not merely a passive bystander that changes alongside aging; rather, it functions as a central hub linking local gut homeostasis to the progressive decline of systemic organ function [125]. On the one hand, age-associated microbial dysbiosis is accompanied by a loss of beneficial commensals and a decline in short-chain fatty acid production, especially butyrate, together with enrichment of pro-inflammatory taxa and broader remodeling of microbial-host metabolic outputs [126]. On the other hand, these alterations directly impair the gut epithelium by reducing mucin and tight-junction integrity, increasing gut permeability, and promoting the translocation of microbial components that fuel systemic inflammation [72, 125]. In parallel, dysbiosis-associated metabolic remodeling affects host serum metabolomic pathways and immune aging, while transfer of young-donor microbiota can partially rejuvenate aged hematopoietic stem-cell function by suppressing inflammation [126]. Moreover, aged microbiota can propagate gut-derived signals to the brain, contributing to gut and brain inflammation, cognitive decline, and behavioral abnormalities through butyrate-FFAR2/3-dependent mechanisms [72]. In other words, gut microbiota dysbiosis does not influence aging through a single linear pathway; instead, it acts in parallel through metabolic, inflammatory, barrier-related, immune, and gut-brain pathways, thereby coupling and reinforcing multiple aging phenotypes within a unified biological network [126].

This network of crosstalk is often initiated by alterations in the gut microecology and the spectrum of microbial metabolites [13]. With advancing age, the decline in SCFAs not only weakens the energy supply to colonic epithelial cells and compromises the maintenance of tight junctions, but also diminishes support for regulatory T-cell differentiation, anti-inflammatory macrophage polarization, and mucus secretion [8, 127, 128]. At the same time, the expansion of potentially pathogenic taxa such as Proteobacteria, the increased burden of lipopolysaccharide (LPS), and disturbances in bile acid and tryptophan metabolism collectively undermine gut barrier integrity and promote the development of a "leaky gut" [129–131]. Once the barrier is disrupted, bacterial components and metabolic toxins can more readily traverse the gut wall and enter the circulation, thereby transforming what was initially a localized disruption of the gut microenvironment into systemic low-grade inflammation and metabolic stress [132]. Thus, gut barrier dysfunction should not be viewed as an isolated event; rather, it represents the combined consequence of microbial compositional shifts and metabolic derangement, while simultaneously serving as the critical gateway through which local imbalance propagates into systemic aging [132].

On this basis, gut-derived inflammation further establishes a positive feedback loop with immunosenescence. LPS and other microbe-associated molecules entering the host can persistently activate TLR4/NF-κB signaling and the NLRP3 inflammasome, thereby promoting the release of pro-inflammatory cytokines such as IL-1β, IL-6, and TNF-α and accelerating the progression of inflammaging [133, 134]. Metabolic dysregulation and oxidative stress, in turn, act as major amplifiers that extend this inflammatory network toward organ functional decline [104, 107]. Dysregulated microbiota-derived metabolites can remodel host glucose and lipid metabolism as well as mitochondrial homeostasis through FXR, TGR5, AhR, AMPK, and related redox pathways [135]. A reduction in SCFAs impairs metabolic adaptability, whereas the accumulation of aberrant bile acids, trimethylamine N-oxide (TMAO), and other toxic metabolites promotes insulin resistance, lipid deposition, vascular aging, and reactive oxygen species (ROS) accumulation [103]. Meanwhile, chronic inflammation further drives immune and tissue cells to generate ROS, leading to DNA, lipid, and protein damage and inducing cellular senescence together with the release of the SASP. SASP, in turn, exacerbates both local and systemic inflammation while continuously damaging the gut barrier and tissue microenvironment [103, 136]. This suggests that metabolic dysregulation, oxidative stress, and inflammaging are not merely parallel processes, but rather integral components of a self-reinforcing loop driven by gut microbiota dysbiosis. In other words, gut microbial imbalance can progressively amplify localized metabolic alterations into functional decline across multiple organ systems [136].

Therefore, from a systems biology perspective, the role of the gut microbiota in aging should not be defined merely as that of a participant in a single pathway, but rather as a pivotal hub linking the local gut microenvironment to systemic aging phenotypes. Its significance lies not only in explaining why aging organisms simultaneously exhibit chronic inflammation, barrier fragility, metabolic abnormalities, and neurofunctional imbalance, but also in clarifying why these phenotypes mutually reinforce one another, undergo cascading amplification, and ultimately culminate in multi-organ functional decline. On this basis, future anti-aging interventions should not be confined to correcting any single terminal phenotype. Instead, they should be centered on the gut microbiota as a core regulatory node, with coordinated strategies aimed at restoring microbial homeostasis, repairing barrier integrity, remodeling immune function, correcting metabolic disturbances, and re-establishing neuroendocrine balance, thereby enabling comprehensive intervention in the systemic aging network.

## Factors affecting the gut microbiota in the elderly

**Fig. 4:**
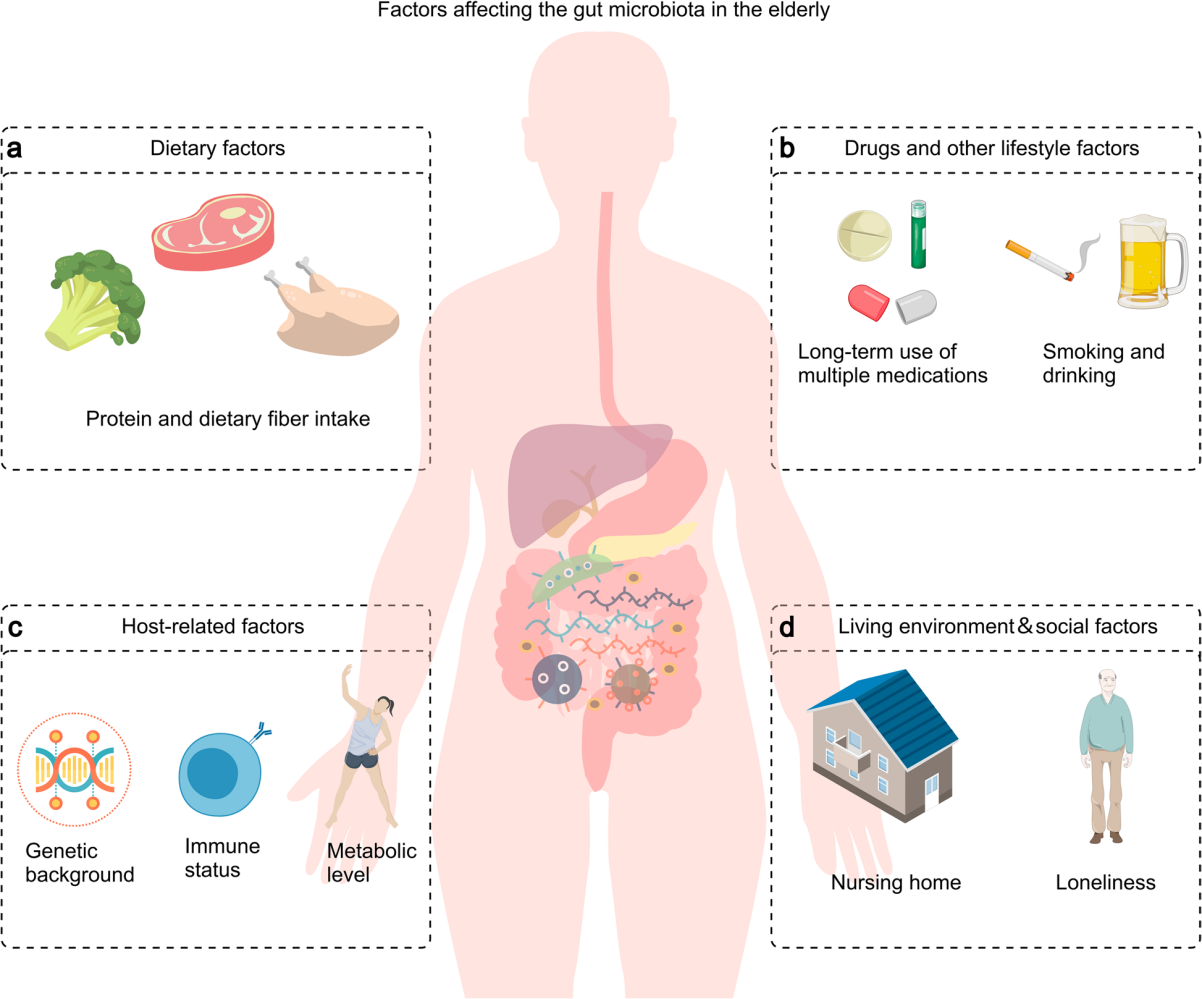
(**a**) Dietary factors, including protein and dietary fiber intake, influence gut microbial composition in the elderly. (**b**) Drugs and lifestyle factors, such as long-term medication use, smoking, and drinking, modulate gut microbiota structure. (**c**) Host-related factors, including genetic background, immune status, and metabolic level, contribute to age-related microbial remodeling. (**d**) Living environment and social factors, including nursing home residence and loneliness, further affect gut microbial diversity and homeostasis

Gut microbiota dysbiosis in older adults does not arise from a single determinant, but rather from the combined influence of intrinsic physiological alterations and external environmental exposures. With advancing age, immune function, metabolic status, and the gut microenvironment undergo progressive changes, while extrinsic factors such as dietary patterns, medication exposure, lifestyle, and living environment continue to shape the composition of the gut microbiota. Consequently, substantial heterogeneity is often observed among older individuals in both gut microbial profiles and their associated health outcomes. To better elucidate the mechanisms underlying age-related alterations in the gut microbiota, the contributing factors can be broadly categorized into two major groups: intrinsic factors and extrinsic factors (Fig. 4).

### Extrinsic factors

Compared with intrinsic determinants, extrinsic factors often exert a more direct influence on the gut microbiota of older adults and, importantly, are more amenable to intervention. Among these, diet represents one of the most critical external regulators of the gut microbial ecosystem. Dietary intake is the most immediate factor shaping the gut microbiota. Dietary fiber serves as a key prebiotic substrate, promoting the growth of beneficial microbes and helping to maintain microbial homeostasis [137–139]. In contrast, dietary insufficiency or imbalanced dietary patterns in older adults can reduce the abundance of beneficial bacteria, including butyrate-producing taxa, and contribute to reduced microbial diversity and frailty-associated dysbiosis [24]. Adherence to a Mediterranean-style diet rich in whole grains, fruits, vegetables, and other high-fiber foods has been shown to significantly enhance gut microbial diversity, enrich health-associated beneficial taxa, reduce inflammatory markers, and improve physical performance and cognitive function in older adults [140, 141]. In addition, animal studies have demonstrated that lifelong caloric restriction of 30%−40% can increase gut microbial diversity in aged mice and shift the relative proportions of Firmicutes and Bacteroidetes toward a more youthful profile, suggesting that moderate dietary interventions may delay age-related microbial dysbiosis [142]. Overall, these findings indicate that dietary modulation remains one of the most practical strategies for preserving microbial homeostasis in later life [142].

Second, older adults frequently require long-term use of multiple medications because of chronic disease, and this has profound implications for the gut microbiota. Broad-spectrum antibiotics can markedly reduce microbial diversity and predispose individuals to drug-resistant infections such as Clostridioides difficile [143–145]. Long-term use of proton pump inhibitors alters gastric acidity, thereby reshaping the gastrogut environment and microbial composition [144]. Nonsteroidal anti-inflammatory drugs may damage the gut mucosa and exacerbate gut permeability, further disrupting microbial ecological balance [146]. Importantly, the effects of medications on the gut microbiota are not uniform [147]. For example, metformin has been reported to selectively increase the abundance of Akkermansia muciniphila and other beneficial taxa, which may contribute to improvements in gut metabolism and inflammatory status [148]. Because polypharmacy is common in older populations, the factors influencing the gut microbiota become increasingly complex, and rational medication use may therefore contribute to the maintenance of microbial homeostasis [149].

At the same time, lifestyle-related factors, including physical activity, psychological status, sleep, smoking, and alcohol consumption, also modulate the gut microbiota. Regular exercise has been shown to increase gut microbial diversity and enrich beneficial bacterial populations, thereby helping to counteract age-related microbial alterations [149, 150]. Better sleep quality is likewise associated with higher gut microbiota richness in older adults [151]. By contrast, smoking and excessive alcohol consumption exert detrimental effects on the gut microbiota: smokers often exhibit a higher proportion of pathogenic bacteria together with a reduction in beneficial taxa, whereas chronic alcohol exposure can damage the gut mucosa and promote the expansion of pro-inflammatory microbial communities [152]. Overall, a healthy lifestyle appears to play an important role in preserving microbial diversity and sustaining a favorable balance of beneficial bacteria in older adults [153, 154].

Finally, living environment and social support may also influence the gut microbiota in later life. Older adults residing long-term in institutional care settings often display significantly lower gut microbial diversity than community-dwelling older individuals, likely owing to factors such as dietary homogenization and reduced physical activity, and their microbial compositions also tend to be more similar to one another [11]. In addition, psychological states such as social isolation and depression may affect microbial homeostasis through the gut-brain axis [155]. These observations suggest that housing conditions, social interaction, and mental health should be considered alongside nutrition, medication use, and physical activity when seeking to preserve a healthy gut microbial ecosystem in old age.

### Intrinsic factors

With advancing age, gut microbial diversity generally declines, accompanied by a reduction in beneficial SCFA-producing bacteria and an expansion of potentially pathogenic microorganisms. These alterations may be closely associated with age-related deterioration in gut structure and function. Although host genetic background may influence the colonization of certain bacterial taxa, twin studies suggest that the overall composition of the gut microbiota is shaped predominantly by extrinsic factors such as diet and environment [152, 156]. By comparison, the contribution of genetic factors to microbial diversity appears to be relatively limited [157]. Another important determinant is immunosenescence: in older adults, decreased mucosal secretion of immunoglobulin A (IgA) and impaired mucosal immune surveillance may permit the excessive proliferation of microbial populations that are normally kept in check, thereby disrupting ecological balance within the gut [158].

Differences in metabolic status and overall health among older individuals also exert a profound influence on the gut microbiota. The concept of a "healthy aging microbiome" has therefore been proposed, whereby healthy long-lived individuals tend to harbor gut microbial communities enriched in anti-inflammatory and beneficial bacteria, whereas frail older adults are more likely to exhibit an increased abundance of pro-inflammatory taxa [155, 159]. In line with this concept, longitudinal and population-based studies suggest that gut microbiome patterns in healthy ageing are associated with better survival, healthier physiological trajectories, and greater interindividual microbial uniqueness [155].

In addition, genetic background may, to some extent, participate in shaping the gut microbiota in later life. Twin studies indicate that the colonization and abundance of certain specific microorganisms may be influenced by host genetic factors; however, overall, genetic determinants generally explain less of the variation in gut microbial structure and diversity than environmental factors [156, 160]. Even so, interindividual differences in genetic background, rate of aging, and burden of underlying disease may still lead to distinct trajectories of microbial evolution during aging [157, 159]. Taken together, the pace of host aging, metabolic health, and genetic variation collectively determine the individual trajectory of gut microbiota remodeling in older adults.

In summary, age-related alterations in the gut microbiota arise from the combined action of multiple factors. Although chronological aging itself plays a central role, the plasticity of the gut microbiota in response to diet and lifestyle offers valuable opportunities for intervention. Through nutritional support, rational pharmacological management, and the adoption of healthy lifestyle practices, it may be possible to partially reverse or at least delay age-associated gut microbial dysbiosis.

## Anti-aging intervention strategies related to gut microbiota

**Fig. 5:**
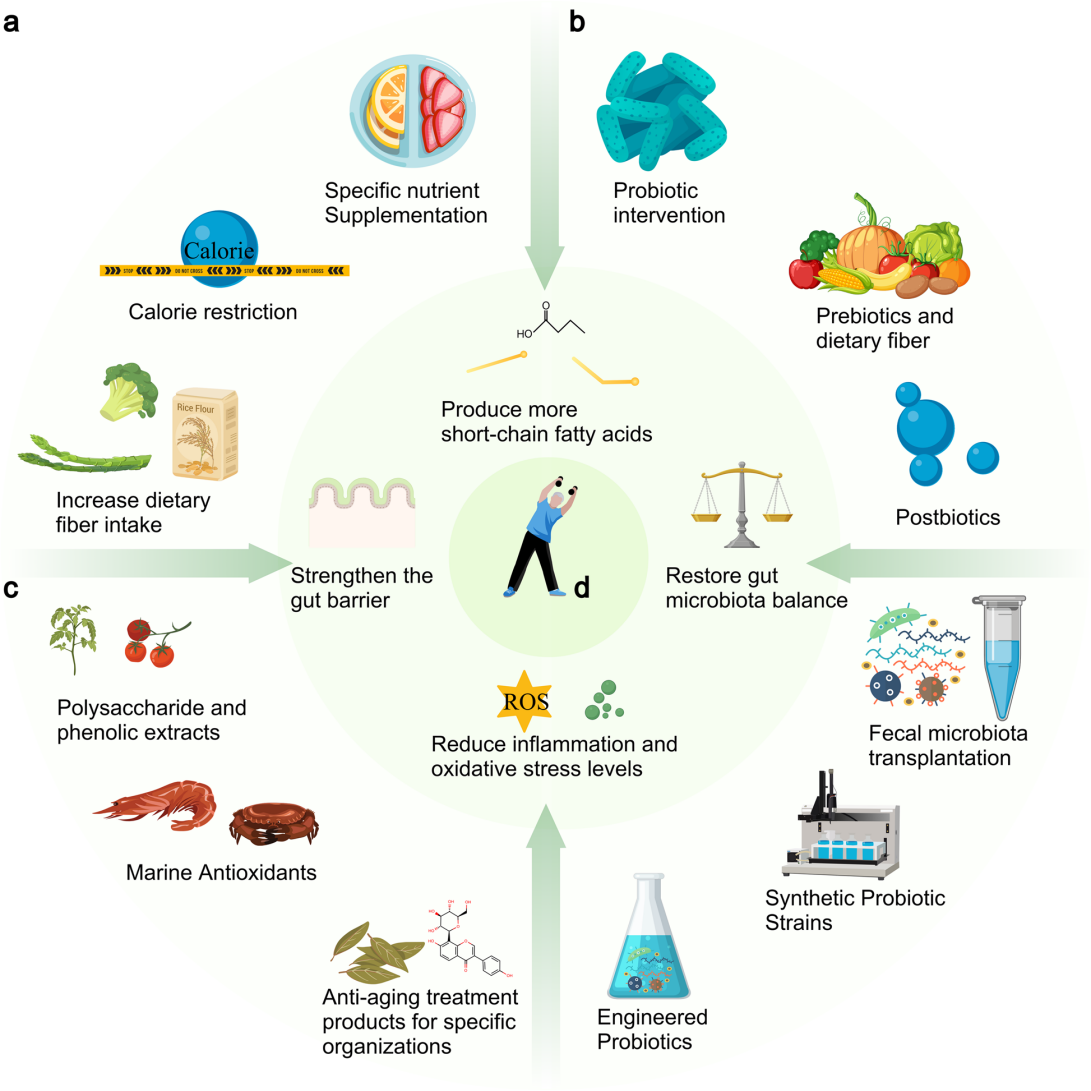
(**a**) Dietary interventions, including calorie restriction, dietary fiber intake, and nutrient supplementation, regulate the gut microbiota during aging. (**b**) Probiotics, prebiotics, postbiotics, fecal microbiota transplantation, and engineered probiotics help restore gut microbial balance. (**c**) Natural products, including polysaccharide, phenolic, and marine antioxidant extracts, modulate microbiota-related aging processes. (**d**) These interventions may alleviate aging by increasing short-chain fatty acid production, strengthening the gut barrier, restoring microbial homeostasis, and reducing inflammation and oxidative stress. ROS: reactive oxygen species

Since the gut microbiota has such an important impact on the aging process, can adjusting or improving the gut microbiota delay aging and promote elderly health? In this section, we will introduce the main current intervention methods targeting the gut microbiota, including dietary interventions, probiotics and prebiotics, fecal microbiota transplantation, and microbiome regulation strategies derived from natural products. It should be noted that many of these strategies are still in the research and experimental stages but show promising prospects (Fig. 5).

### Dietary and caloric intervention

"Dietary intervention" is the most economical and direct way to regulate the gut microbiota. As discussed above, age-related microbial imbalance in older adults is strongly influenced by dietary patterns. Therefore, adjusting the dietary structure is expected to reshape a healthier gut microenvironment.

First, increasing dietary fiber intake is widely considered beneficial for the gut microbiota of the elderly. Diets rich in fermentable dietary fiber and resistant starch can promote the proliferation of beneficial fermentative bacteria, producing more short-chain fatty acids [161]. Studies have shown that increasing dietary fiber and plant-based food intake for six weeks in frail elderly individuals can significantly raise the levels of butyrate and beneficial bacteria like Bacteroides in their feces, while reducing inflammatory markers [162]. Elderly people who consistently follow a Mediterranean-style high-fiber diet tend to have a more diverse gut microbiota composition and a higher proportion of anti-inflammatory bacteria, which is associated with better cognitive function and lower risk of chronic diseases. In contrast, a high-fat, high-sugar, low-fiber "Western diet" exacerbates dysbiosis in the elderly and accelerates the onset of aging-related chronic diseases [163, 164]. Therefore, elderly nutritional guidance should emphasize adequate dietary fiber intake, which not only supports gut health but also improves systemic inflammation and metabolic status through microbiota metabolic products [165].

Secondly, calorie restriction (CR), a classic intervention for lifespan extension, may have some benefits mediated by the gut microbiota [166]. It has been previously mentioned that lifelong CR in mice can maintain a more "youthful" microbial composition [167]. In human populations, moderate calorie restriction has also been found to have a positive impact on the microbiota [168]. A two-year randomized controlled trial showed that adults undergoing a 25% calorie restriction had a reduced ratio of Firmicutes to Bacteroidetes in their gut, which is speculated to be associated with a healthier metabolic phenotype. Although strict long-term calorie restriction is generally unrealistic or unsafe for most elderly individuals, alternative practices such as intermittent fasting are gradually gaining attention in older populations. Some preliminary studies indicate that mild intermittent fasting can increase gut bacteria that produce proteolytic enzymes and reduce gut and systemic inflammation levels [169]. Additionally, a high-quality high-protein diet is beneficial for sarcopenia in the elderly, but high protein intake may reduce fiber consumption, so balance should be maintained [170, 171]. In the future, methods such as specialized formulated foods and gut microbial transplantation may be considered to achieve both adequate nutrition and beneficial microbiota construction [171].

Furthermore, specific nutrient supplementation can selectively regulate the microbiota. For example, supplementation with omega-3 polyunsaturated fatty acids has been found to increase the abundance of the mucosal probiotic Akkermansia, alleviating the adverse effects of a high-fat diet on the microbiota. Supplementing multiple vitamins and minerals can compensate for dietary deficiencies in the elderly, indirectly maintaining sufficient substrates for microbial metabolism [172]. It should be noted that supplementation with common prebiotics in the elderly is also part of dietary intervention, such as inulin and fructooligosaccharides(FOS), which selectively promote the growth of bifidobacteria and lactobacilli [165, 172]. Studies have shown that elderly volunteers who took daily inulin experienced an increase in gut bifidobacteria numbers, accompanied by a reduction in digestive discomfort symptoms [173]. The mechanism of prebiotics lies in providing the fermentable substrates needed by beneficial bacteria, enhancing their competitiveness, thereby improving microbiota structure and increasing beneficial metabolites such as SCFAs [165].

In summary, a reasonable diet should serve as the fundamental strategy for elderly people to maintain the health of their gut microbiota. Personalized nutrition plans not only benefit traditional nutritional health but also exert anti-aging effects through the gut microbiota, known as the "hidden organ." Before other medical or supplementary interventions, dietary optimization is the most cost-effective and side-effect-free method. Of course, for very elderly individuals who already have malnutrition or require special care, dietary adjustments should be gradually made under the guidance of clinical nutritionists to avoid gastrogut intolerance caused by excessive restriction or sudden increases in fiber.

### Probiotics, prebiotics, and postbiotics

Probiotics are generally defined as active microbial preparations that can colonize the host and provide health benefits after ingestion, commonly including bifidobacteria and lactobacilli [174]. Prebiotics refer to indigestible nutritional components that selectively promote the growth and activity of one or several probiotics, such as inulin and galacto-oligosaccharides. Postbiotics generally refer to functional products generated by probiotic metabolism or inactivated probiotics themselves [175]. These three are important strategies for regulating the microbiota used in anti-aging and are collectively regarded as essential components of microbiome therapy.

Probiotic intervention has been demonstrated by multiple animal experiments to improve aging phenotypes. For example, supplementation of Lactobacillus acidophilus in accelerated aging mice can reduce their inflammation factor levels and improve aging signs such as hair loss [176, 177]. In humans, evidence for probiotics is also accumulating. A randomized placebo-controlled trial administering Lactobacillus plantarum HEAL9 capsules to elderly individuals over 70 years old for 4 weeks found that fecal calprotectin levels significantly decreased in the probiotic group, suggesting a reduction in local gut inflammation. Although systemic inflammation markers such as serum CRP did not show significant decreases, the probiotic group showed slight improvements in some cognitive function tests. Another trial targeting elderly individuals with mild cognitive impairment showed that after 12 weeks of supplementation with Lactobacillus paracasei and Bifidobacterium breve, subjects improved in memory scores compared to the control group, with the presumed mechanism related to reduction of gut and systemic inflammation levels [176]. Additionally, probiotics are effective for certain gut disorders in the elderly, such as preventing antibiotic-associated diarrhea and Clostridioides difficile infections [177]. Currently, commercially available probiotic preparations for the elderly are mostly multi-strain combinations aimed at comprehensively improving digestive and immune functions. It should be noted that the safety of probiotics in very old or severely immunocompromised individuals still requires attention [176, 177]. Overall, probiotic supplementation moderately improves inflammatory status in the elderly, but its exact effect on delaying aging still requires larger-scale clinical validation.

Prebiotics and dietary fiber: Prebiotics indirectly improve the microbiota by creating an environment favorable for the growth of probiotics. In fact, prebiotics such as inulin can also be used as supplements. Studies have shown that adding FOS to elderly populations can significantly increase the number of bifidobacteria in feces and reduce fecal pH, helping to inhibit the growth of putrefactive bacteria [165]. Prebiotics often require a certain dosage to be effective, but excessive doses may cause discomfort such as bloating; therefore, clinical application should be progressive [172]. Besides traditional prebiotics, some polyphenolic compounds also have prebiotic properties [178, 179]. They are not absorbed in the upper small intestine, but are metabolized by microbes after reaching the colon, promoting the proliferation of beneficial bacteria and inhibiting pathogenic bacteria. Polyphenolic prebiotics have shown antioxidant and anti-inflammatory effects, which may benefit the cardiovascular and nervous systems of the elderly [180]. The advantage of prebiotics lies in their high safety and good compliance, making them a suitable intervention form for elderly people.

Postbiotics: Considering that some elderly people may have difficulty tolerating large amounts of live bacteria or high fiber, directly supplementing microbial metabolites has become another strategy. For example, oral sodium butyrate capsules can partially mimic the effects of butyrate-producing bacteria [181]. In animal experiments, exogenous butyrate can reduce inflammation and oxidative stress levels in aged mice [182]; in vitro studies also confirmed that butyrate can enhance the gut barrier by activating the PPAR-γ pathway [183]. Regarding human trials, studies on butyrate formulations for inflammatory bowel disease provide insights for elderly inflammatory gut permeability. Besides SCFAs, other postbiotics such as short-chain peptides and surface molecules are also under development. For instance, 3-phenyl lactic acid has been reported to extend healthy lifespan in model organisms [184]. Postbiotic therapy is still in its early stages but has the advantages of defined components and ease of standardization; in the future, it may be used synergistically with probiotics.

Probiotics, prebiotics, and postbiotics represent different levels of microbiome interventions, ranging from introducing live bacteria to providing "feed" and directly supplying the products [185]. For the elderly population, an ideal approach may be multi-faceted, such as a diet rich in natural prebiotics, combined with regular intake of fermented dairy products or supplements containing probiotics, supplemented by key metabolic products. It is important to emphasize that microbiome interventions usually show slow effects, requiring continuous use along with lifestyle improvements to demonstrate benefits. At the same time, effective strains and dosages may vary among individuals, and in the future, personalized "precision probiotics" schemes are expected to be developed based on individual microbiome characteristics. Currently, some scholars have proposed administering specific probiotics or metabolites according to the functional deficiencies of the microbiota in the elderly, for example, focusing on supplementing acid-producing bacteria for those lacking SCFAs. This precise microecological therapy awaits further research and clinical trial validation.

### Fecal microbiota transplantation and emerging microbiome therapies

Fecal microbiota transplantation (FMT) is a treatment that involves transplanting fecal microbiota from a healthy donor into the patient's intestine to restore a normal microbial community. FMT has shown significant efficacy in gut diseases such as Clostridium difficile infection. In recent years, interesting animal studies have suggested that FMT may also be used to intervene in the aging process.

Classic experiments include transplanting the microbiota of young animals into aged animals. In one study, scientists transplanted the gut microbiota of young fish into middle-aged African killifish; the fish receiving the young microbiota lived longer and showed improved age-related behavioral decline compared to the non-transplanted group [186]. This "microbiome rejuvenation" experiment indicates that, in the long term, regularly "refreshing" the gut ecology of aged hosts with microbiota from young donors holds potential to delay the deterioration of certain aging markers [186].

Currently, the exploration of FMT for anti-aging in humans is still at a very preliminary stage [187]. Some clinical trials of FMT targeting elderly frailty syndrome are being planned. For example, small-scale studies plan to transplant the microbiota from healthy young volunteers to very old frail elderly individuals to observe changes in inflammatory markers and muscle strength [188]. There are also FMT trials aimed at elderly people with mild cognitive impairment, hoping to improve their gut microbiota to delay cognitive decline. However, so far, publicly reported human trial data is very limited, and the efficacy and safety of FMT in anti-aging remain inconclusive. It should be considered that elderly individuals often have multiple underlying diseases and frailty, so directly conducting FMT may carry risks of infection or other side effects. In addition, donor selection and the persistence of microbiota colonization are also issues [189]. One approach is to first optimize the FMT protocol—for example, donor screening to include specific anti-inflammatory microbiota, and appropriate gut preparation of recipients before transplantation. Recent studies suggest using encapsulated microbiota preparations for FMT to reduce invasiveness [190]. In summary, FMT has theoretical appeal in the field of anti-aging, but its actual implementation may require more time for research and ethical review.

Besides FMT, other emerging microbiome therapies are also worth mentioning. For example, synthetic probiotic consortia are created by combining multiple key strains from healthy individuals into colonizable microbial capsules. Compared to traditional single-strain probiotics, this "mixed-consortium drug" is closer to FMT but with defined components and great potential. Some have tried treating ulcerative colitis with mixtures of dozens of strictly pathogen-free gut bacteria and achieved success [191]. In the future, it may be possible to design microbial consortium formulations specifically targeting elderly inflammation or metabolic syndrome. Another example is phage therapy, which uses specialized bacteriophages to eliminate pathogenic bacteria and regulate microbiota composition [62]. In cases where elderly patients are colonized by Clostridioides difficile or drug-resistant E. coli, phages are expected to precisely eradicate these harmful bacteria without disturbing other symbiotic microbes. Additionally, engineered probiotics are also a hot area. Researchers have constructed genetically modified lactic acid bacteria and E. coli Nissle strains that can release therapeutic molecules within the gut. For example, engineered bacteria that continuously synthesize anti-inflammatory factors like IL-10 after colonization can alleviate colitis in mice [62]; theoretically, engineered bacteria secreting antioxidants or free radical-neutralizing substances could be developed to reduce inflammatory aging. Furthermore, recently proposed paraprobiotics are protein-polysaccharide complexes secreted by probiotics and attached to the bacterial surface, which can modulate immunity without live bacteria. If these paraprobiotics can be extracted as oral formulations, they might avoid the safety concerns of live bacterial preparations in immunocompromised elderly individuals.

Although the above emerging therapies sound promising, many obstacles must be overcome before they can be applied in clinical anti-aging practice, including regulatory approval, individual response variability, and long-term safety. However, they represent the future direction of microbiome medicine. It is foreseeable that microecological therapy will evolve from the current "rough transplantation" to more precise and customized approaches. In the future, elderly patients may receive specific microbial populations or genetically engineered bacteria supplements based on their individual microbiome deficiencies, thus targeting and correcting aging-related disorders. In this process, current methods such as FMT provide us with valuable experience and insights.

### Plant extracts and natural products

Active compounds abundant in many traditional herbs and plants have been found to regulate gut microbiota and possess antioxidant and anti-inflammatory effects, holding potential value for anti-aging. Unlike probiotics mentioned above, these natural products often do not directly supplement microorganisms but exert their effects indirectly by modifying the gut environment or providing substrates for microbial metabolism to influence the microbiota. Below, we briefly illustrate several representative types of natural products and their mechanisms of action and discuss their application prospects (Table 1).

**Table 1:** Representative natural products targeting gut microbiota in aging-related intervention studies

Natural products	Preclinical evidence	Clinical evidence status	Clinical considerations and recommendations
Cistanche Polysaccharides	In D-galactose-induced aging mice, CDPS improved cognition, restored gut microbial homeostasis, and reduced oxidative stress, supporting a microbiota–gut–brain mechanism	No directly relevant human trial linking Cistanche–gut microbiota–aging outcomes was identified in this targeted search	Design: randomized, placebo-controlled trial in older adults with mild cognitive impairment (MCI) or cognitive frailty for 12–24 weeks. Hypothesis: CDPS increases beneficial taxa and SCFAs, reduces inflammaging, and improves memory and executive function
Lycopene	In female CD-1 mice with D-gal-induced aging, lycopene attenuated memory and behavioral deficits by mediating the microbiota–SCFAs–gut–brain axis [192]	A small human intervention study reported dose-dependent gut microbiota changes after lycopene intake, but participants were moderately obese adults rather than elderly aging cohorts, and aging endpoints were not primary outcomes [193]	Design: double-blind RCT in older adults with subjective cognitive decline or frailty. Hypothesis: lycopene remodels SCFA-producing microbes, improves barrier function and systemic oxidative stress, and slows cognitive and frailty progression
Polygonatum polysaccharides	Recent work in D-gal-induced aging mice showed renoprotective effects of Polygonatum sibiricum polysaccharides with linked gut microbiota and metabolomics changes; earlier work in 5xFAD mice also showed cognition-related microbiota remodeling [194]	No direct human study connecting Polygonatum polysaccharides–microbiota–aging outcomes was identified in this targeted search	Design: pilot RCT in older adults with sarcopenia or early cognitive decline. Hypothesis: PSP improves host metabolic resilience by enriching beneficial taxa, reinforcing barrier integrity, and reducing neuroinflammation or muscle catabolism
Astaxanthin	In D-gal-induced aging mice, astaxanthin and Haematococcus pluvialis alleviated liver injury through the gut–liver axis, with improved antioxidant status and microbial remodeling [195]	A randomized double-blind placebo-controlled trial reported cognitive benefit of astaxanthin-containing supplementation in people with MCI, but gut microbiota was not evaluated, so this is only indirect translational support [196]	Design: older adults with MCI or metabolic frailty; include metagenomics, SCFA,bile acids, cognition, liver enzymes, and oxidative-stress markers. Hypothesis: astaxanthin improves cognition or metabolic aging partly through gut microbiota remodeling
Oleuropein	Oleuropein modulated gut microbiota and improved metabolic phenotypes in experimental T2DM; rat work also suggested altered enteric bacterial flora. These support microbiota relevance, but the evidence is not aging-specific [197]	No direct human study linking oleuropein–gut microbiota–aging clinical outcomes was identified in this targeted search	Design: older adults with metabolic syndrome or inflammaging. Hypothesis: oleuropein shifts gut microbial composition, improves barrier integrity and glucose-lipid metabolism, and thereby attenuates metabolic aging
Astragalus Polysaccharide	APS alleviated S. aureus-disrupted mastitis by regulating gut microbiota and SCFAs metabolism; other recent animal studies also support microbiota-mediated barrier and immune benefits. Aging specificity is moderate rather than strong	No direct aging-focused human microbiota trial of APS was identified in this targeted search	Design: older adults with low-grade inflammation, impaired gut barrier, or post-infectious frailty. Hypothesis: APS increases SCFA-producing taxa, strengthens gut barrier, and reduces systemic inflammatory burden
Puerarin	Puerarin delayed mammary gland aging by regulating gut microbiota and inhibiting p38MAPK signaling; other studies support microbiota-related barrier and anti-inflammatory effects [198]	No direct clinical evidence linking puerarin–microbiota–aging was identified in this targeted search	Design: peri-postmenopausal women or older women with mammary or estrogen-related aging phenotypes. Hypothesis: puerarin improves microbial diversity, lowers SASP-associated inflammation, and delays tissue aging
Coix seed extract	Recent animal studies show coix seed oil can restore gut microbiota balance, repair barrier injury, and improve inflammation; some work also suggests benefit in arthritis-associated functional decline. These are aging-related but not classic anti-aging models [199]	No direct human anti-aging microbiota study was identified in this targeted search	Design: older adults with osteoarthritis plus metabolic and inflammatory burden. Hypothesis: coix-derived products reduce gut-driven inflammation and improve pain, mobility, and microbiota diversity
Brown algae polyphenols	Brown macroalgae polyphenol extracts restored SCFAs and modulated gut microbiota in diabetic rodents; a 2025 study also suggested anti-aging potential of Hizikia fusiforme polyphenol–polysaccharide complex with microbiota modulation [200]	No direct human microbiota-aging trial was identified in this targeted search	Design: older adults with metabolic syndrome or cognitive frailty. Hypothesis: seaweed polyphenols increase SCFA production, improve oxidative and immune homeostasis and attenuate metabolic or neurocognitive aging
Monolaurin	In high-fat-diet models, glycerol monolaurate attenuated metabolic syndrome and modulated gut microbiota; high-dose administration also significantly remodeled microbiota and metabolic outcomes. This supports translational relevance but not aging specificity [201]	No direct human study linking monolaurin–gut microbiota–aging was identified in this targeted search	Design: older adults with obesity, NAFLD risk, or metabolic frailty. Hypothesis: monolaurin reduces gut pathogen burden and endotoxemia, improving hepatic and metabolic aging markers

Polysaccharide and phenolic extracts, some derived from medicinal and edible plants, have attracted much attention for their effects on the gut microbiota and aging axis. For example, polysaccharides extracted from desert Cistanche (CDPS) have been confirmed to improve cognitive function in a D-galactose-induced premature aging mouse model. Mechanistically, CDPS increased the relative abundance of beneficial gut bacteria such as Bacteroidetes and Firmicutes in aged mice, reduced harmful bacteria levels, and restored microbial diversity; meanwhile, it elevated neurotrophic factors in the mouse brain and decreased the oxidative stress marker MDA content [202]. This suggests that Cistanche polysaccharides exert anti-aging effects by modulating the gut-brain axis. Another example is lycopene, a carotenoid abundant in tomatoes, which also helps with brain aging. A study showed that daily gavage of lycopene to aging mice significantly improved their spatial memory compared to controls [203]. Mechanistically, lycopene restored gut microbiota diversity in elderly mice, increased the abundance of short-chain fatty acid-producing bacteria, and reduced pro-inflammatory bacteria. The concentrations of acetate and butyrate in the feces of the lycopene-treated group increased; these SCFAs may cross the blood–brain barrier to improve synaptic plasticity and mitochondrial function in the brain, thus alleviating cognitive impairment [203]. Notably, research also demonstrated the microbiota dependency of this effect through fecal microbiota transplantation: transplanting fecal microbiota from lycopene-treated mice to untreated mice conferred similar cognitive improvements [203]. Additionally, polysaccharides extracted from the Chinese medicinal herb Polygonatum (PSP) have been reported to protect against aging-related skeletal muscle atrophy. Cell experiments showed that PSP activated the PI3K/Akt/mTOR pathway in muscle cells and suppressed muscle atrophy factors MuRF1 and Atrogin-1, thereby reducing muscle protein degradation [203]. Although it is not yet fully clear whether PSP acts through the gut microbiota, mice treated with PSP showed some optimization of gut microbiota, suggesting that PSP might intervene in muscle aging via bidirectional regulation of the gut-muscle axis. Overall, polysaccharide and phenolic plant extracts generally possess both prebiotic and antioxidant effects; they improve the microbial composition as dietary components while directly scavenging free radicals or regulating host signaling—either by themselves or through their metabolites—thereby delaying aging.

Marine antioxidants, certain pigments and polyphenols derived from marine sources also show potential in delaying aging. For example, astaxanthin is a red carotenoid found in shrimp, crabs, and Haematococcus pluvialis, with strong antioxidant capacity [195]. In a study using a D-galactose-induced aging mouse model, intervention with astaxanthin or Haematococcus pluvialis extract for 8 weeks resulted in significant improvement in antioxidant markers in the mice's liver [195]. Compared to the model group, the astaxanthin-treated mice showed increased liver SOD and GSH-Px activity and decreased MDA levels, indicating reduced oxidative damage. Meanwhile, the diversity of their gut microbiota also improved, suggesting that astaxanthin may protect the aging liver via the gut-liver axis by directly scavenging free radicals to protect liver cells and by promoting the growth of beneficial bacteria to reduce endotoxin production, thus protecting liver function through a dual mechanism. Additionally, brown algal polyphenols, abundant in seaweed, have similar antioxidant and anti-inflammatory effects and have been used in dietary supplement development [195, 204]. These marine natural products warrant further study for their potential in intervening in age-related liver damage and skin aging.

Some natural products have been studied for aging-related diseases in specific tissues. For example, oleuropein, derived from olive leaves, has been shown to inhibit canine mammary tumor cells. Research shows that oleuropein dose-dependently inhibits the proliferation of canine mammary tumor cells and induces their apoptosis, with mechanisms related to inhibition of the PI3K/Akt pathway and regulation of pro-apoptotic proteins Bax/Bcl-2 [205]. In a mouse model of breast cancer, this may delay the malignant transformation of mammary tissue and is considered a potential drug for anti-breast aging. However, it should be noted that the doses used in cell experiments are relatively high, and the clinical significance remains uncertain. Astragalus polysaccharides (APS), derived from the traditional Chinese medicine Astragalus, have been studied in a mouse model of mammary gland inflammation induced by Staphylococcus aureus [205]. APS was found to reduce bacterial load and inflammatory infiltration in mammary tissue and alter the gut microbiota composition, specifically increasing the abundance of butyrate-producing Butyricicoccus. The increase in probiotics led to elevated levels of acetate and butyrate in feces, which, through blood circulation, alleviated inflammation and oxidative stress in the mammary gland. This suggests that APS may act through the gut-mammary axis, regulating the microbiota, promoting SCFA production, and reaching the mammary gland via blood to exert anti-inflammatory and antioxidant effects、 alleviating aging-related changes in mammary tissue. Puerarin, an isoflavone extract from the leguminous plant Pueraria lobata, has been shown to improve atrophy and fibrosis of mammary acini in aging female mice and reduce senescence-associated β-galactosidase staining [198]. Its mechanisms include inhibiting the p38MAPK inflammatory pathway in mammary tissue, alleviating mitochondrial damage, and indirectly exerting effects by modulating gut microbiota diversity [198]. In the puerarin-treated mouse group, the proportion of Firmicutes increased while Proteobacteria decreased, indicating a healthier microbial composition. It is hypothesized that puerarin metabolites produced by gut microbiota in vivo may have hormone-like or anti-inflammatory activities, thereby protecting mammary tissue and delaying aging.

It is important to emphasize that although many natural products have shown promising anti-aging effects in animal models, there is still a gap before they can be clinically applied. Firstly, most studies remain at the cellular or rodent stage without human trials to support them, so their efficacy and safety are uncertain. Secondly, the doses used in mice often correspond to extremely high human doses, limiting practical application. For example, the effective dose of lycopene in mice may require consuming dozens of tomatoes daily for humans. Thirdly, natural products are complex in composition, and the bioavailability of active ingredients in the human body is limited; for instance, polyphenols are often transformed in the intestines and have low absorption rates, making it challenging to enhance their targeted effects. Additionally, the metabolism of the same phytochemical by gut microbiota can vary greatly among individuals, leading to variability in therapeutic effects. Lastly, the long-term safety of some herbal extracts needs monitoring, such as potential burdens on the liver and kidneys or effects on the metabolism of other drugs.

Therefore, a cautious yet optimistic attitude should be maintained regarding natural products for anti-aging. They provide many potentially effective new molecules and mechanisms, but more clinical evidence-based research is needed before they can be transformed into products usable by the elderly. In the future, these natural compounds might be combined with microbiome science, for example, by using synthetic biology techniques to introduce beneficial genes into gut symbiotic bacteria so that they continuously produce low doses of natural products in the body, achieving long-lasting and safe intervention. This approach is already being explored and is worth looking forward to. In summary, natural products, as an adjunct to microbiota intervention, may offer beneficial supplements to healthy aging under the concept of "medicine and food sharing the same origin," but their application must be based on scientific evidence and safety assessments.

Overall, plant extracts and natural products have shown considerable promise in delaying organismal aging and ameliorating age-related functional decline by reshaping gut microbial composition, restoring microbial metabolic function, and alleviating chronic inflammation and oxidative stress. However, the current body of evidence remains dominated by animal studies and mechanistic investigations, while robust clinical evidence is still relatively limited. Moreover, substantial heterogeneity persists across studies with respect to target populations, evaluation metrics, follow-up duration, and the direction of observed outcomes. Therefore, for these intervention strategies to achieve genuine clinical translation, their efficacy, consistency, and boundaries of applicability must be further validated in higher-quality human studies.

### Hierarchical evaluation framework for anti-aging research on the gut microbiota

In this review, our evaluation of research on the gut microbiota and aging does not rely on a framework that merely ranks studies according to design hierarchy or judges their value solely on the basis of whether positive results were obtained. Instead, we adopt a three-tier evaluative framework that progresses from observable phenomena to mechanistic interpretation and ultimately to practical application, namely, the aging phenotype level, the mechanistic explanation level, and the clinical translation level.

At the first level, the aging phenotype level, the primary concern is whether alterations in the gut microbiota are associated with key manifestations of aging, including frailty, cognitive decline, chronic low-grade inflammation, metabolic dysfunction, reduced physical performance, and the broader state of healthy aging. At the second level, the mechanistic explanation level, the focus shifts to whether these associations can be plausibly explained by pathways such as changes in microbial metabolites, impairment of the gut barrier, dysregulation of immune and inflammatory homeostasis, heightened oxidative stress, and neuroendocrine disturbance, and whether causal support can be established through animal experiments, fecal microbiota transplantation, and molecular pathway validation. At the final level, the clinical translation level, the central question is whether microbiota-related interventions can be translated into stable, reproducible, and medically meaningful benefits in human populations.

This evaluative pathway is well aligned with the prevailing direction of contemporary aging research. Aging can no longer be adequately defined by a single molecular indicator; rather, it must be assessed through the combined consideration of functional phenotypes and molecular mechanisms. At the same time, modern aging research emphasizes the importance of establishing a continuous chain of evidence from basic discovery to clinical application by using biomarkers capable of capturing short-term biological alterations while also predicting long-term clinical outcomes such as frailty, morbidity, and mortality [206, 207]. In parallel, recent cohort and multi-omics studies have identified chronic inflammation and gut microbiota remodeling as measurable aging-associated processes that are closely linked to health status, frailty risk, and survival [208]. Together, these findings provide a strong empirical basis for evaluating the gut microbiota within a continuous “phenotype–mechanism–translation” paradigm [209].

Viewed through this framework, the evidence summarized throughout this review can be reinterpreted in a more coherent evaluative sequence. Evidence describing the decline in microbial diversity in older adults, the disruption of core microbial taxa, and the altered profile of microbial metabolites primarily belongs to the aging phenotype level. The significance of this evidence lies in revealing the distinct microecological features that differentiate “healthy aging” from “unhealthy aging.”

Evidence related to immunosenescence, gut barrier disruption, metabolic dysregulation, oxidative stress, neuroendocrine regulation, animal experiments, fecal microbiota transplantation, metabolite supplementation, and molecular pathway validation further contributes to the mechanistic explanation level. These findings suggest that the gut microbiota is not merely an epiphenomenon of aging, but may actively participate in the cascading amplification from local microecological imbalance to systemic functional decline through mechanisms such as SCFA depletion, increased LPS burden, aberrant bile acid and tryptophan metabolism, and the positive feedback loop between inflammation and oxidative stress.

On this basis, dietary interventions, probiotics, prebiotics, postbiotics, fecal microbiota transplantation, and natural product-based modulation enter the domain of evidence at the clinical translation level. At this stage, evaluation should not remain confined to the question of whether these interventions alter microbial composition; rather, it should focus more critically on whether they improve clinically relevant endpoints, including frailty, cognitive function, muscle strength, metabolic status, quality of life, and disease risk.

It should be emphasized that negative findings do not necessarily indicate that gut microbiota-targeted interventions are “ineffective”; rather, they may reflect the marked endpoint specificity and stratification dependence of such interventions [210]. The PROMOTe randomized controlled trial showed that, although all participants received branched-chain amino acid supplementation and recommendations for resistance exercise, the additional administration of a prebiotic did not improve the primary physical performance endpoint, namely the time required to complete five chair rises. However, it did improve composite cognitive scores and reduced the number of errors in the paired-associate learning test [210]. These findings suggest that the potential benefits of microbiota-based interventions may initially emerge in specific functional domains rather than manifest uniformly across all clinical endpoints.

Accordingly, evidence synthesis in the field of gut microbiota and aging should avoid simplistic judgments based solely on “positive results” or on study design hierarchy alone, and should instead emphasize the complementarity of different forms of evidence: cross-sectional studies are useful for identifying candidate microbiota-phenotype associations [208], longitudinal cohorts for validating stability and predictive value, randomized controlled trials for confirming modifiability and domain-specific clinical benefit, and animal experiments together with fecal microbiota transplantation studies for strengthening causal inference and elucidating biological mechanisms [211].

Therefore, rather than simply claiming that gut microbiota intervention has already become a mature anti-aging strategy, we emphasize its dual significance as both an integrative therapeutic target and a stratification tool. On the one hand, the gut microbiota links multiple domains of aging, including immunity, barrier integrity, metabolism, and neuroendocrine regulation, thereby providing a more systemic point of intervention for healthy aging than approaches focused on a single organ or pathway. On the other hand, microbial composition, metabolic function, and host response characteristics may serve as important foundations for future population stratification, efficacy prediction, and treatment monitoring. Although the clinical translation of microbiome research is currently accelerating, its genuine integration into geriatric practice will still depend on mechanistic consistency, the clinical relevance of endpoints, long-term safety, individualized stratification strategies, and the reproducibility of outcomes. For this reason, the evaluative hierarchy adopted in this review—“aging phenotype–mechanistic explanation–clinical validation”—more faithfully reflects the actual developmental trajectory of gut microbiota research, from phenomenological association to mechanistic interpretation and ultimately to clinical application.

## Future perspectives and challenges

Although research on the gut microbiota and aging has expanded rapidly in recent years, progressing from descriptive analyses of microbial composition to mechanistic investigations involving metabolic regulation, immune inflammation, gut barrier integrity, and the gut-brain axis, the field as a whole remains at a stage characterized by an abundance of correlational evidence but insufficient causal evidence, fragmented local mechanisms but inadequate systemic integration, and active basic research but relatively slow clinical translation. The most pressing questions for future investigation are therefore no longer broad inquiries such as whether the gut microbiota changes during aging, but rather several more fundamental and penetrating scientific issues: whether gut microbiota dysbiosis is merely a concomitant phenomenon of host aging, an upstream driving force, or part of a self-amplifying feedback loop in which both processes mutually reinforce one another; which key microbial taxa, microbial metabolites, and host signaling axes play decisive roles at different stages of aging; and how the gut microbiota evolves from a localized microecological system into a systemic regulatory node that links immune, metabolic, barrier, and neuroendocrine networks in aging. Only by constructing a clearer chain of evidence around these questions can gut microbiota research truly advance from phenomenological association to mechanistic explanation and, ultimately, to translatable intervention.

First, the most prominent limitation of current population-based studies lies in the insufficient capacity of existing study designs to support causal inference. Most available studies remain predominantly cross-sectional, typically relying on one-time comparisons between young and older individuals, healthy older adults and frail older adults, or centenarians and ordinary elderly populations, and then inferring from these observations that certain microbial characteristics are associated with aging status. Although such designs are valuable for identifying differences, they are inherently unable to determine whether microbiota alterations precede or follow the emergence of aging phenotypes, nor can they distinguish true driving factors from passive consequences secondary to disease, medication use, dietary modification, or organ functional decline. In addition, older populations are highly heterogeneous and are frequently affected by underlying diseases, long-term polypharmacy, altered dietary patterns, reduced physical activity, disrupted sleep rhythms, and differences in social environment, all of which can independently and substantially shape the gut microbiota. Accordingly, future cohort studies should move beyond static comparison toward dynamic longitudinal tracking. It will be essential to establish multicenter longitudinal follow-up systems spanning middle-aged individuals, older adults, those in the prefrail and frail stages, and even long-lived populations. Through the continuous collection of fecal, blood, urine, and clinical phenotypic data, and by systematically recording changes in biological age, frailty indices, inflammatory status, cognitive function, metabolic indicators, and organ function, such studies may identify microbial trajectories that truly precede aging phenotypes and possess predictive value.

Second, important technical limitations persist in the current literature, particularly the tendencies to emphasize composition over function, averages over spatial organization, and single time points over dynamic change. A substantial proportion of studies still relies primarily on 16S rRNA sequencing to describe shifts in microbial structure. While this approach can identify which taxa increase or decrease, it offers only limited insight into what these microorganisms are actually doing. Aging, however, is unlikely to be determined solely by changes in microbial abundance; rather, it is more plausibly shaped by microbial functional output and the manner in which this output is coupled to host responses. Future research must therefore move beyond single-layer microbial profiling toward integrated multi-omics approaches that combine metagenomics, metatranscriptomics, metabolomics, proteomics, host transcriptomics, immune phenotyping, and clinical endpoints. Particular attention should be given to identifying aging-related functional modules, such as reduced short-chain fatty acid biosynthetic capacity, aberrant bile acid transformation profiles, dysregulated tryptophan–indole metabolism, increased endotoxin burden, and remodeling of oxidative stress–related metabolic networks. Instead of broadly stating that “beneficial bacteria decline while harmful bacteria increase,” future studies should seek to determine which functional deficits emerge earliest, which metabolite groups serve as the principal intermediaries linking microbial alterations to organ aging, and which host receptors or signaling pathways act as the critical hubs through which these effects are amplified. In other words, the central objective should shift from constructing a taxonomic atlas of aging to building a functional atlas of aging.

Third, future mechanistic research must move beyond the current limitation of describing individual modules in isolation and instead establish models of cross-system crosstalk and cascading amplification. In the preceding sections, immunosenescence, gut barrier disruption, metabolic dysregulation, oxidative stress, and neuroendocrine regulation have been discussed separately; however, the more important question for future investigation is why these processes deteriorate simultaneously in the aging organism, and which of them serve as the initiating events, the amplifiers, and the terminal effectors. One conceptual framework that warrants particular emphasis proposes that aging and environmental exposures first disrupt microbial homeostasis, resulting in a decline in short-chain fatty acid–producing bacteria and an increase in pro-inflammatory microbes and their metabolites. This is followed by a reduction in gut barrier integrity, allowing microbial-associated molecules such as lipopolysaccharide (LPS) to translocate more readily into the circulation. Subsequently, innate immune pathways and inflammasome signaling are persistently activated, thereby driving chronic low-grade inflammation and oxidative stress. Chronic inflammation, in turn, further impairs mitochondrial function, neuroendocrine homeostasis, and multiorgan metabolic regulation, ultimately generating a vicious cycle in which immunosenescence, metabolic aging, and organ functional decline mutually reinforce one another. Future studies should therefore focus on this continuous chain of events—microbiota dysbiosis, barrier disruption, inflammatory amplification, metabolic and neuroendocrine imbalance, and multiorgan aging—and define the key cell types, molecular mediators, and temporal windows at each stage. Only by integrating these mechanisms into a coherent causal framework, rather than treating them as parallel observations, can the scientific significance of the gut microbiota as a central regulatory node in systemic aging be more fully elucidated.

Fourth, the advancement of model systems will determine whether mechanistic research in this field can achieve genuine depth. Future investigations should move beyond conventional correlation analyses and routine phenotypic observations in animal models, and instead increasingly incorporate germ-free animals, defined microbial colonization models, humanized microbiota transplantation, gut organoids, organ-on-a-chip platforms, single-cell sequencing, and spatial omics technologies. Germ-free or antibiotic-depletion models can help determine whether specific microbial communities are sufficient to alter host aging phenotypes; fecal microbiota transplantation and reconstitution with defined consortia can further distinguish the effects of the overall microbial community from those of individual strains. Organoid and organ-on-a-chip systems, meanwhile, provide powerful platforms for precisely dissecting how microbial metabolites influence epithelial renewal, tight junction protein expression, stem cell homeostasis, and inflammatory responses. Single-cell and spatial omics approaches hold particular promise for revealing how immune cells, epithelial cells, glial-like cells, and stromal cells in the aging intestine respond to microbial signals within their local microenvironment. Truly valuable mechanistic research in the future should not merely demonstrate that a given microbe or metabolite is associated with aging, but should clarify which cell types it targets, through which receptors it acts, which signaling pathways it modulates, and at what stage of aging its effects are most critical.

Fifth, clinical translation remains both the most compelling and the most challenging direction in this field. Although studies on probiotics, prebiotics, synbiotics, dietary pattern modification, fecal microbiota transplantation, and natural product–based interventions are steadily increasing, the field as a whole continues to face substantial limitations, including small sample sizes, short follow-up periods, inconsistent endpoint definitions, inadequate population stratification, and insufficient reproducibility. Future interventional research must therefore shift from generalized supplementation toward precision modulation. On the one hand, participants should be stratified according to baseline microbial composition, dietary habits, inflammatory status, biological age, and comorbid disease burden, so as to determine which individuals are most likely to benefit from probiotic supplementation, which are more responsive to dietary fiber–based microbial remodeling, and which may derive greater benefit from postbiotics, targeted metabolite supplementation, or engineered microbial delivery systems. On the other hand, clinical trial endpoints should not remain limited to microbial diversity or a small number of inflammatory markers, but should instead incorporate more clinically meaningful composite outcomes, such as frailty indices, sarcopenia-related measures, cognitive performance, fall risk, improvement in metabolic syndrome, hospitalization rates, and even healthspan-related endpoints. At the same time, greater attention must be paid to long-term safety, patient adherence, dose–response relationships, and the durability of therapeutic effects after treatment discontinuation. In particular, for fecal microbiota transplantation, engineered microbes, and combination microecological products, ethical oversight, safety regulation, and quality control frameworks must be strengthened in parallel.

Finally, future research should place greater emphasis on healthy aging, rather than focusing exclusively on pathological aging. At present, many studies concentrate on microbiota alterations in the context of age-related disorders such as Alzheimer’s disease, metabolic syndrome, atherosclerosis, and frailty syndrome. Yet the populations most capable of illuminating the fundamental basis of longevity and high-quality aging are often those who retain substantial functional reserve even at advanced age. Centenarians, healthy long-lived individuals, and those resistant to frailty may harbor microbial ecological patterns and host response profiles with genuine protective significance. If future studies can investigate pathological aging and healthy aging in parallel, and construct not only a spectrum of microbiota-associated risk factors but also a profile of microbiota-associated protective factors, this will more effectively support the development of proactive strategies aimed at promoting healthy aging.

In summary, the next stage of research on the gut microbiota and aging should move beyond cross-sectional descriptions of group differences toward approaches that are longitudinal, function-oriented, causal, and precision-based. It should also progress from the exploration of isolated mechanisms to the integration of multiple physiological systems, and from empirical intervention toward stratified clinical translation. What is truly worth anticipating in the future is not merely the identification of a few “longevity-associated microbes” or the development of several “anti-aging probiotics,” but the establishment of a microecological theoretical framework capable of explaining the heterogeneity of aging, predicting aging trajectories, and guiding precise intervention. At that point, the gut microbiota will no longer be regarded as a mere accompanying variable in aging research, but rather as a central regulatory hub linking host immune homeostasis, metabolic networks, barrier function, and neuroendocrine regulation, thereby providing a new theoretical foundation and intervention pathway for delaying functional decline and promoting healthy aging.

## Data Availability

Data sharing is not applicable to this article as no datasets were generated or analyzed during the current study.
